# An Anti-Inflammatory 2,4-Cyclized-3,4-Secospongian Diterpenoid and Furanoterpene-Related Metabolites of a Marine Sponge *Spongia* sp. from the Red Sea

**DOI:** 10.3390/md19010038

**Published:** 2021-01-16

**Authors:** Chi-Jen Tai, Chiung-Yao Huang, Atallah F. Ahmed, Raha S. Orfali, Walied M. Alarif, Yusheng M. Huang, Yi-Hsuan Wang, Tsong-Long Hwang, Jyh-Horng Sheu

**Affiliations:** 1 Doctoral Degree Program in Marine Biotechnology, National Sun Yat-sen University, Kaohsiung 80424, Taiwan; chijentai@g-mail.nsysu.edu.tw; 2 Department of Marine Biotechnology and Resources, National Sun Yat-sen University, Kaohsiung 80424, Taiwan; betty8575@yahoo.com.tw; 3 Department of Pharmacognosy, College of Pharmacy, King Saud University, Riyadh 11451, Saudi Arabia; rorfali@ksu.edu.sa; 4 Department of Pharmacognosy, Faculty of Pharmacy, Mansoura University, Mansoura 35516, Egypt; 5 Department of Marine Chemistry, Faculty of Marine Sciences, King Abdulaziz University, Jeddah 21589, Saudi Arabia; welaref@kau.edu.sa; 6 Department of Marine Recreation, National Penghu University of Science and Technology, Magong, Penghu 88046, Taiwan; yusheng@gms.npu.edu.tw; 7 Tropical Island Sustainable Development Research Center, National Penghu University of Science and Technology, Magong, Penghu 88046, Taiwan; 8 Graduate Institute of Natural Products, College of Medicine, Chang Gung University, Taoyuan 33302, Taiwan; d0901501@cgu.edu.tw (Y.-H.W.); htl@mail.cgu.edu.tw (T.-L.H.); 9 Research Center for Chinese Herbal Medicine, Research Center for Food and Cosmetic Safety, Graduate Institute of Health Industry Technology, College of Human Ecology, Chang Gung University of Science and Technology, Taoyuan 33303, Taiwan; 10 Department of Anesthesiology, Chang Gung Memorial Hospital, Taoyuan 33305, Taiwan; 11 Graduate Institute of Natural Products, Kaohsiung Medical University, Kaohsiung 80708, Taiwan; 12 Department of Medical Research, China Medical University Hospital, China Medical University, Taichung 404333, Taiwan

**Keywords:** Red Sea sponge, *Spongia*, seco-spongian diterpenoid, isoprenoid-derived amide

## Abstract

Chemical investigation of a Red Sea *Spongia* sp. led to the isolation of four new compounds, i.e., 17-dehydroxysponalactone (**1**), a carboxylic acid, spongiafuranic acid A (**2**), one hydroxamic acid, spongiafuranohydroxamic acid A (**3**), and a furanyl trinorsesterpenoid 16-*epi*-irciformonin G (**4**), along with three known metabolites (−)-sponalisolide B (**5**), 18-nor- 3,17-dihydroxy-spongia-3,13(16),14-trien-2-one (**6**), and cholesta-7-ene-3β,5α-diol-6-one (**7**). The biosynthetic pathway for the molecular skeleton of **1** and related compounds was postulated for the first time. Anti-inflammatory activity of these metabolites to inhibit superoxide anion generation and elastase release in *N*-formyl-methionyl-leucyl phenylalanine/cytochalasin B (fMLF/CB)-induced human neutrophil cells and cytotoxicity of these compounds toward three cancer cell lines and one human dermal fibroblast cell line were assayed. Compound **1** was found to significantly reduce the superoxide anion generation and elastase release at a concentration of 10 μM, and compound **5** was also found to display strong inhibitory activity against superoxide anion generation at the same concentration. Due to the noncytotoxic activity and the potent inhibitory effect toward the superoxide anion generation and elastase release, **1** and **5** can be considered to be promising anti-inflammatory agents.

## 1. Introduction

Marine sponges have been considered to be an important source for the discovery of structurally diverse bioactive secondary metabolites [1]. Many natural products from sponges have been shown to exhibit a variety of biological activities, such as antimicrobial [2,3,4,5], antiviral [6,7,8], antiprotozoal [8,9,10], cytotoxic [6,11,12,13], anti-inflammatory [14,15,16], antioxidant [4,17,18], immunosuppressive [1,19,20], and antifeedant [21,22,23]. The genus *Spongia* (Spongidae) has been chemically investigated since 1971 [24] and the studies have led to the discovery of a series of furanoterpenes [24,25,26], spongian diterpenoids [27,28,29,30,31,32], scalarane sesterterpenoids [33,34,35], sesquiterpene quinones [36,37], along with other kinds of metabolites, for example, sterols [38,39,40] and macrolides [41].

**Figure 1:**
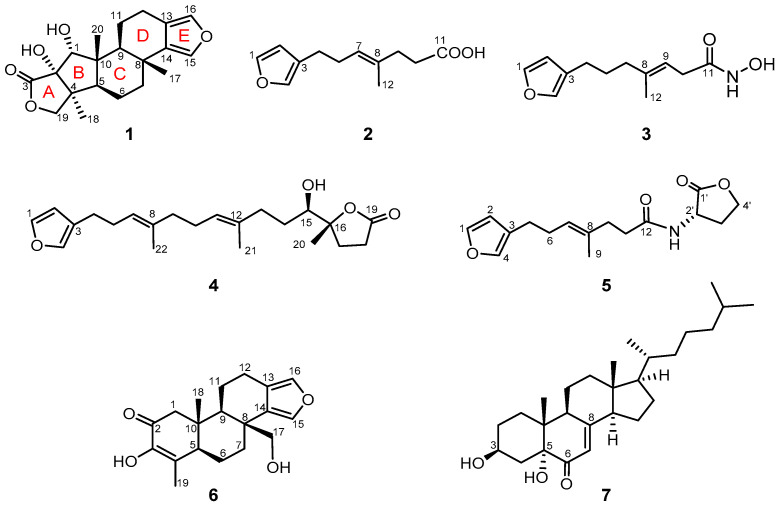
Structures of compounds **1**–**7** isolated from a Red Sea *Spongia* sp.

We report, herein, the chemical investigation of an unidentified *Spongia* species inhabiting along the eastern coast of the Red Sea. This study afforded four new natural products including a rare A-ring contracted diterpenoid, 17-dehydroxysponalactone (**1**), a C_12_ carboxylic acid, spongiafuranic acid A (**2**); a C_12_ hydroxamic acid, spongiafuranohydroxamic acid A (**3**); and a furanyl trinorsesterpenoid, 16-*epi*-irciformonin G (**4**); along with three known metabolites, (−)-sponalisolide B (**5**) [42], 18-nor-3,17-dihydroxyspongia-3,13(16),14-trien-2-one (**6**) [43], and cholesta-7-ene-3β,5α-diol-6-one (**7**) [40] (Figure 1 and Supplementary Materials Figures S1–S35 for **1**–**5**). Furthermore, in order to discover bioactive lead compounds, assays for the anti-inflammatory activity of the isolated compounds by inhibition of the superoxide anion generation and elastase release in *N*-formyl-methionyl-leucyl phenylalanine/cytochalasin B (fMLF/CB)-induced human neutrophils, and the cytotoxicity of these compounds against three tumor cell lines, murine leukemia (P388), human bile duct carcinoma (HuCCT), and human colon adenocarcinoma (DLD-1), and a human dermal fibroblast (CCD-966SK) cell line were undertaken. Compounds **1** and **5** were shown to exhibit the promising anti-inflammatory activity.

## 2. Results and Discussion

**Figure 2:**
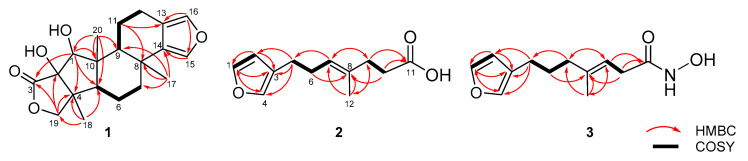
Selected ^1^H-^1^H COSY and HMBC correlations for **1**, **2,** and **3**.

**Scheme 1:**
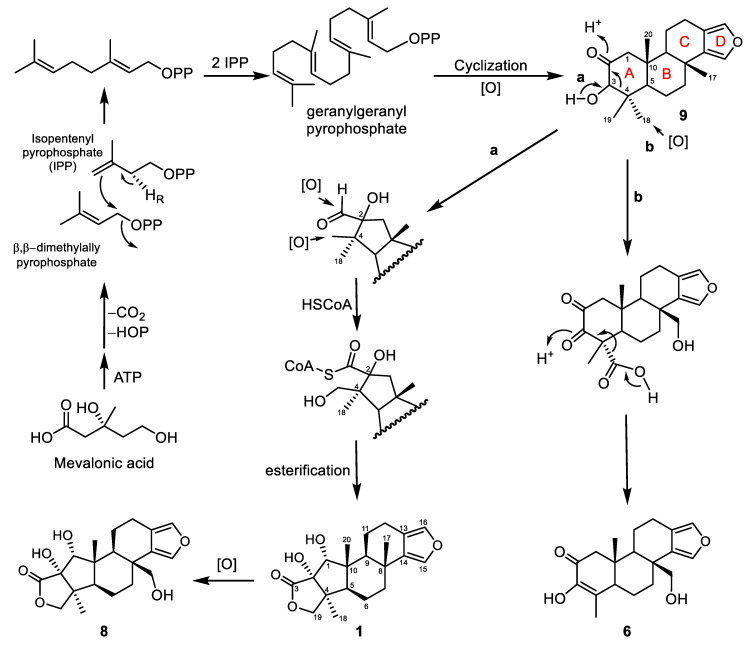
Plausible biosynthetic pathway of **1**, **6,** and related metabolites.

Compound **1** was obtained as a white powder. Its molecular formula C_20_H_26_O_5_ was established by the molecular ion peak at *m/z* 369.1672 [M + Na]^+^ in the HRESIMS, consistent with eight degrees of unsaturation. The IR spectrum showed absorptions of hydroxyl (3455 and 3401 cm^−1^) and lactone carbonyl (1752 cm^−1^) functionalities. The ^13^C NMR spectroscopic data of **1** exhibited 20 carbon signals (Table 1), which were assigned by the assistance of DEPT spectrum showing thirteen carbon signals of a diterpene, including three ring-juncture methyls (δ_C_ 26.9, 22.6, and 14.0; δ_H_ 1.24, 1.14, and 0.84) and a 3,4-disubstituted furan ring (δ_C_ 137.1, CH; 134.8, CH; 136.8, C; and 119.6, C and δ_H_ 7.06, 1H, br s and 7.09, 1H, br s) [30,31,35,44]. On the basis of the number of unsaturations, **1** was, thus, suggested to be a pentacyclic 3,4-disubstituted furan diterpenoid. The NMR spectroscopic data of **1** and 2D NMR corraltions (Figure 2) were similar to those of the previously described sponalactone (**8**) [30], except that a hydroxymethyl in **8** was replaced by a methyl at C-8 in **1**. Compound **1** also possesses the same B, C, and D rings as **9** [32] (Scheme 1).

**Figure 3:**
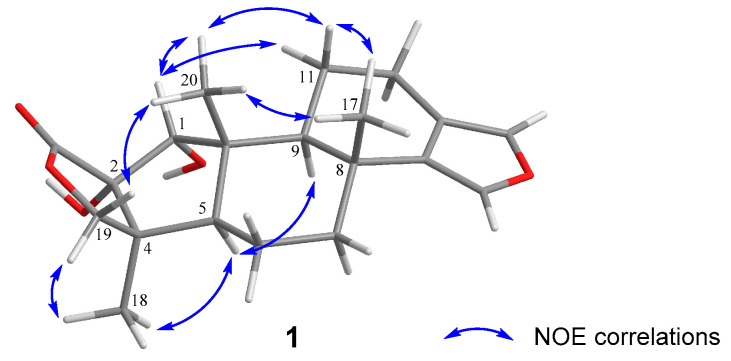
Selected nuclear Overhauser effect (NOE) correlations for **1**.

The relative and absolute configurations of **1** were established on the basis of nuclear Overhauser effect (NOE) correlation analysis (Figure 3) and by comparison of the observed NOE correlations with those of the related compounds [30,31], the observed pyridine-induced solvent shifts [45], and biogenetic consideration. The NOESY spectrum of **1** showed NOE correlations of H_3_-17/H_3_-20 and H-5/H-9, depicting the 5R*,8R*,9S*,10R*-configuration. H-1 displayed NOE interactions with the *β*-oriented H_3_-20 and H-11α (δ_H_ 1.68, m), indicating the α-orientation of the H-1. Furthermore, the NOE correlations of H-5/H_3_-18, H_3_-18/H-19*α* (δ_H_ 3.92) and H-19*β* (δ_H_ 4.37)/H_3_-20 disclosed the *α*- and *β*-orientations of H_3_-18 and the γ-lactone ring, respectively, and the *α*-orientation of the hydroxyl at C-2, accordingly. The analysis of the pyridine-induced deshielding effect of the axial hydroxy groups was also employed to support the configuration of **1**. Therefore, the significant pyridine-induced downfield shifts (∆δ = δ_CDCl3_ − δ_C6D5N_) exerted on H-5 (∆δ_H_ = −0.24 ppm) could only be approached when 1-OH was axially oriented on the same α-face of the molecule. Also, H_3_-18 exhibited pyridine-induced downfield shift (∆δ_H_ = −0.14 ppm) due to the vicinal effect of 2-OH, which should be *syn* to H_3_-18 [45]. On the basis of the above findings, we propose that **1** can be derived from an intermediate spongian **9**, which was biosynthesized from the mevalonic acid pathway, after oxygenation of the six-membered ring A and a subsequent ring contraction and formation of a five-membered carbocycle, as illustrated in Scheme 1.

Metabolite **2** was isolated as a colorless oil. Its molecular formula was determined to be C_12_H_16_O_3_ from the HREIMS (*m/z* 231.0992 [M + Na]^+^), indicating the four degrees of unsaturation. The IR spectrum displayed the absorptions of carboxylic acid (3105–2857 and 1708 cm^–1^) and olefin (1654 cm^−1^). The NMR data (Table 2) showed the presence of a monosubstituted furan ring (δ_C_ 142.5, CH; 138.8, CH; 111.0, CH; and 124.7, C; δ_H_ 7.34, 7.20, and 6.27, each 1H, s) [24,25,26,42], a trisubstituted olefin (δ_C_ 124.7, CH; δ_H_ 5.22, 1H, s), a methyl (δ_C_ 15.9; δ_H_ 1.61, 3H, s) and a carbonyl group (δ_C_ 180.0, C). Other ^1^H NMR signals in the shielded region (δ_H_ 2.25–2.47, 8H) were attributable to four methylene groups, as depicted from the COSY (Figure 2) correlations. The methylene protons H_2_-6 (δ_H_ 2.25, dt, *J* = 7.6, 7.2 Hz, 2H) was found to be further correlated with the olefinic proton (δ_H_ 5.22, dd, *J* = 7.2, 7.2 Hz, H-7) in **2**. The detailed analysis of HMBC correlations (Figure 2) resolved the carbon positions of the furan ring, olefinic double bond, and the carboxyl group to be at C-1‒C-4, C-7/C-8, and C-11, respectively. Furthermore, the methyl group was positioned at C-8. The furanyl H-2 (δ_H_ 6.27, s), H-4 (δ_H_ 7.20, s), and the olefinic proton H-7 (δ_H_ 5.22, dd, *J* = 7.2, 7.2 Hz, 2H) displayed HMBC correlations with the *sp*^3^ carbon C-5 (δ_C_ 24.8, CH_2_), and H_3_-12 (δ_H_ 1.61, s) showed HMBC correlations with C-7 (δ_C_ 124.7, CH) and C-9 (δ_C_ 34.2, CH_2_), while the signal of H_2_-9 (δ_H_ 2.32, dd, *J* = 7.6, 7.6 Hz, 2H) was found to be correlated with the carboxyl carbon (C-11, δ_C_ 180.0). Moreover, the NOE correlations observed for H_3_-12 with H_2_-6 but not with H-5 and the chemical shift of C-12 (δ_C_ < 20 ppm) assigned the *E-*configuration of the 7,8-double bond [46]. Therefore, **2** was determined to be a furanotrinorsesquiterpenoid carboxylic acid with the structure of (*E*)-7-(furan-3-yl)-4-methylhept-4-enoic acid. The literature search showed that this compound had been prepared as a synthetic intermediate during the total syntheses of the furanosesquiterpenoids and dendrolasins [42,47], however, its NMR data had not been reported. Therefore, this is the first report of **2** as a natural product, with the NMR data assigned and reported for the first time.

Metabolite **3** exhibited almost the same NMR data as those of **2** (Table 2) from C-1 to C-6, with the carbon chemical shifts of the trisubstituted double bond (δ_C_ 139.7, C and 115.5, CH; δ_H_ 5.34, dd, *J* = 6.0, 6.0 Hz, 1H) and the carbonyl group (δ_C_ 176.1, C) in **3** showing significant differences of ∆δ_C_ −6.0, +9.2, and −3.9 ppm as compared with those of the corresponding carbons in **2**, respectively. As illustrated by ^1^H-^1^H COSY correlations (Figure 2), the double bond has been isomerized from the C-7/C-8 position in **2** to the C-8/C-9 position in **3**. However, the IR spectrum displayed the absorptions of the hydroxyl and NH groups (3407‒2858 cm^–1^), carbonyl group (1705 cm^–1^), and olefin (1634 cm^–1^) functionalities. Furthermore, the HREIMS *m/z* 246.1098 [M + Na]^+^ established the molecular formula of **3** to be C_12_H_17_NO_3_ and the chemical shift of the carbonyl group (176.1 ppm), showing that a hydroxamic acid moiety [48,49,50,51] replaced a carboxylic acid group at C-11 in **3**.

**Figure 4:**
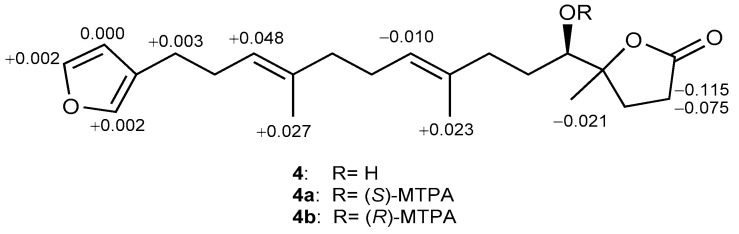
^1^H NMR chemical shift differences Δδ (δS − δR) in ppm for α-methoxy-α-(trifluorome- thyl)-phenylacetyl (MTPA) esters of **4**.

**Figure 5:**
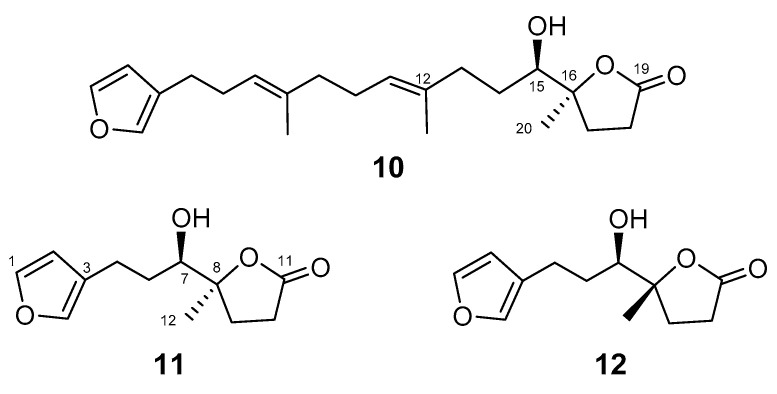
Structures of known compounds **10**–**12**.

Compound **4** was isolated as a colorless oil, [α]${}_{D}^{25}$ +4.4 (*c* 0.74, CHCl_3_). The ESIMS and NMR spectroscopic data (Table 3) established the molecular formula C_22_H_32_O_4_ for **4**. The IR absorptions 3432, 1769, and 1647 cm^–1^ revealed the presence of hydroxyl, carbonyl, and olefin functionalities, respectively. Moreover, it was found that the NMR data of **4** was the same as those of irciformonin G (**10**) [52] in all aspects except for those at positions 17 and 18−20 (Table 4), proposing **4** as an isomer of **10**. By using Mosher’s method [53,54], the 15*R* absolute configuration in **4** was established based on the calculated ∆δ_H_ (δ*_S_* − δ*_R_*) values of protons neighboring C-15 of (*S*)- and (*R*)-α-methoxy-α-(trifluorome- thyl)-phenylacetyl (MTPA) esters **4a** and **4b**, respectively (Figure 4). After the assignment of the 15*R* configuration, the ^13^C NMR data of C-15 to C-20 of **4** were further compared with the corresponding data of irciformonin G (**10**), (+)-sponalisolide A (**11**), and 8-*epi*-(+)-sponalisolide A (**12**) [42] of known absolute configurations (Table 4 and Figure 5). The 15*R*,16*R*-configuration of **4** was, thus, confirmed as those of the 7*R*, 8*R* configured **12,** while **10** and **11** possessed the same configurations (*R*,*S*) at the corresponding asymmetric carbons. From the above findings, compound **4** was, thus, identified as 16-*epi*-irciformonin G.

(−)-Sponalisolide B (**5**) was isolated as a colorless oil, [α]${}_{D}^{25}$ −8.5 (*c* 0.34, CHCl_3_). Through detailed analysis of NMR spectroscopic data (Table 3), in particular two-dimensional (2D) NMR correlations, the structure of **5** was established to be identical to that of the known (‒)-sponalisolide B [42]. However, the coupling constants and spin-spin splitting patterns of the proton H_2_-6 (δ_H_ 2.25, dt, 2H, *J* = 7.5, 7.0 Hz at 500 MHz in CDCl_3_) were wrongly assigned. We, herein, reanalyzed the spectrum and provided the correct NMR data for **5**.

With the aim of discovering bioactive compounds from these isolates, the cytotoxic activities of the isolated compounds **1**−**7** against the proliferation of three cancer cell lines including murine leukemia (P388), human bile duct carcinoma (HuCCT), and human colon adenocarcinoma (DLD-1), and a human dermal fibroblast cell line (CCD-966SK) were evaluated, using the Alamar Blue assay [55,56]. The results indicated that none of the tested metabolites exhibited cytotoxic activity (IC_50_ > 20 μg/mL)**.**

The anti-inflammatory activities of compounds **1**−**7** on inhibition of superoxide anion (O_2_^−^) generation and elastase release in the fMLF/CB-stimulated human neutrophils [57,58,59] were also evaluated. The results (Table 5) showed that **1** exhibited potent activity to inhibit the superoxide anion generation (91.38 ± 2.91%) and elastase release (90.29 ± 7.71%) at 10 μM, with the IC_50_ values of 3.37 ± 0.21 and 4.07 ± 0.60 μM, respectively. Compound **5** was also found to display significant inhibitory activity against the superoxide anion generation (IC_50_ = 5.31 ± 1.52 μM), and the percentage of inhibition was 67.12 ± 6.00% at 10 μM. Due to the noncytotoxic character and the potent activity toward the superoxide anion generation and elastase release, **1** and **5** can be considered to be the promising anti-inflammatory agents.

## 3. Materials and Methods

### 3.1. General Procedures

Measurements of optical rotations and IR spectra were carried out on a JASCO P-1020 polarimeter and FT/IR-4100 infrared spectrophotometer (JASCO Corporation, Tokyo, Japan), respectively. ESIMS and HRESIMS were performed on a Bruker APEX II (Bruker, Bremen, Germany) mass spectrometer. The NMR spectra were recorded on a Varian 400MR FT-NMR at 400 and 100 MHz for ^1^H and ^13^C, respectively or a Varian Unity INOVA500 FT-NMR at 500 and 125 MHz for ^1^H and ^13^C, respectively (Varian Inc., Palo Alto, CA, USA). Silica gel or reversed-phase (RP-18, 230–400 mesh) silica gel was used for column chromatography and analytical thin-layer chromatography (TLC) analysis (Kieselgel 60 F-254, 0.2 mm, Merck, Darmstadt, Germany), respectively. Isolation and purification of compounds by high-performance liquid chromatography (HPLC) were achieved using an Hitachi L-2455 HPLC apparatus (Hitachi, Tokyo, Japan) equipped with a Supelco C18 column (250 × 21.2 mm, 5 μm, Supelco, Bellefonte, PA, USA).

### 3.2. Animal Material

The sponge *Spongia* sp. was collected during March 2016, off the Red Sea Coast at Jeddah, Saudi Arabia (21^o^22′11.08″ N, 39 ^o^06′56.62″ E). A voucher sample (RSS-1) has been deposited at the Department of Pharmacognosy, College of Pharmacy, King Saud University, Saudi Arabia.

### 3.3. Extraction and Separation

The *Spongia* sp. was collected and freeze-dried. The freeze-dried material (550 g dry wt) was minced and extracted exhaustively with EtOAc/MeOH/CH_2_Cl_2_ (1:1:0.5) (3 × 10 L). The solvent-free extract was suspended in water and partitioned with CH_2_Cl_2_, EtOAc, and then *n*-BuOH saturated with water to obtain CH_2_Cl_2_ (18.47 g), EtOAc (0.782 g), and *n*-BuOH (1.0 g) fractions. The CH_2_Cl_2_ fraction was chromatographed over silica gel column, using EtOAc in *n*-hexane (0% to 100%, stepwise), to yield 12 fractions (F1–F12). F6 (1.21 g), eluted with *n*-hexan/EtOAc (1:1), was re-chromatoraphed over a RP-18 column using MeOH in H_2_O (50% to 100%, stepwise) to give 15 subfractions (F6-1 to F6-15). F6-5 (83.0 mg), F6-8 (85.2 mg), F6-11 (21.1 mg), and F6-14 (23.5 mg) were purified on RP-18 HPLC separately, using MeOH/H_2_O (1.4:1), CH_3_CN/H_2_O (1:1.7), MeOH/H_2_O (1.5:1), and CH_3_CN/H_2_O (1.6:1), in order, to afford **2** (55.5 mg) from F6-8, **6** (6.2 mg) from F6-5, **1** (10.2 mg) from F6-11, and **4** (7.4 mg) from F6-14. F7 (1.1 g), eluted with *n*-hexane/EtOAc (1:3), was isolated using RP-18 silica gel column chromatography and MeOH in H_2_O (50% to 100%, stepwise) as a mobile phase to result in 20 subfractions (F7-1 to F7-20). F7-4 (16.1 mg) and F7-6 (25.7 mg) were further separated on RP-18 HPLC, using CH_3_CN/H_2_O (1:1.7) and (1:2.5), separately, to afford **3** (4.6 mg) from F7-4, **5** (9.1 mg) and **7** (4.3 mg) from F7-6.

#### 3.3.1. 17-Dehydroxysponalactone (**1**)

White powder, [α]${}_{D}^{25}$ +27.7 (*c* = 0.71, CHCl_3_); IR (neat) ν_max_ 3455, 3401, 2962, 2927, 2864, 1752, 1663, 1455, 1387, 1186, 1150, 1111, 1060, 1019, 890.0, and 757 cm^–1^; ^1^H NMR (500 MHz, CDCl_3_); and ^13^C (125 MHz, CDCl_3_) data, see Table 1. ESIMS *m/z* 369 [M + Na]^+^; ^1^H NMR (C_5_D_5_N, 400 MHz) δ_H_ 7.37 (1H, br s, H-16), 7.26 (1H, br s, H-15), 4.44 (1H, d, *J* = 9.6 Hz, H-19), 4.30 (1H, br s, H-1), 3.94 (1H, d, *J* = 9.6 Hz, H-19), 2.66 (1H, m, H-12), 2.60 (1H, m, H-12), 2.26 (1H, m, H-9), 2.14 (1H, d, *J* = 11.5 Hz, H-5), 2.12 (1H, m, H-7), 1.76 (1H, m, H-11), 1.67 (1H, m, H-6), 1.60 (1H, m, H-11), 1.57 (1H, m, H-6), 1.56 (1H, m, H-7), 1.28 (3H, s, H_3_-18), 1.24 (3H, s, H_3_-17), 0.94 (3H, s, H_3_-20); ^13^C NMR (C_5_D_5_N, 100 MHz) δ_C_ 181.0 (C, C-3), 138.0 (CH, C-15), 136.0 (C, C-14), 135.7 (CH, C-16), 120.5 (C, C-13), 84.3 (C, C-2), 82.7 (CH, C-1), 74.4 (CH_2_, C-19), 57.0 (CH, C-5), 47.8 (CH, C-9), 47.6 (C, C-4), 47.0 (C, C-10), 40.9 (CH_2_, C-7), 35.1 (C, C-8), 27.4 (CH_3_, C-17), 23.8 (CH_3_, C-18), 20.9 (CH_2_, C-11), 20.4 (CH_2_, C-12), 18.9 (CH_2_, C-6), 14.5 (CH_3_, C-20). HRESIMS *m/z* 369.1672 [M + Na]^+^ (calcd for C_20_H_26_O_5_Na, 369.1673).

#### 3.3.2. Spongiafuranic Acid A (**2**)

Colorless oil, IR (neat) ν_max_ 3105, 2920, 2918, 2857, 1708, 1654, 1500, 1446, 1386, 1298, 1210, 1163, 1024, and 874 cm^–1^; ^1^H NMR (400 MHz, CDCl_3_); and ^13^C (100 MHz, CDCl_3_) data, see Table 2. ESIMS *m/z* 231 [M + Na]^+^. HRESIMS *m/z* 231.0994 [M + Na]^+^ (calcd for C_12_H_16_O_3_Na, 231.0997).

#### 3.3.3. Spongiafuranohydroxamic Acid A (**3**)

Colorless oil, IR (neat) _max_ 3407, 3252, 2918, 2858, 1704, 1634, 1442, 1372, 1298, 1205, 1136, 1027, and 963 cm^–1^; ^1^H NMR (400 MHz, CDCl_3_); and ^13^C (100 MHz, CDCl_3_) data, see Table 2. ESIMS *m/z* 246 [M + Na]^+^. HRESIMS *m/z* 246.1098 [M + Na]^+^ (calcd for C_12_H_17_NO_3_Na, 246.1100).

#### 3.3.4. 16-Epi-Irciformonin G (**4**)

Colorless oil, [α]${}_{D}^{25}$ +4.4 (*c* 0.74, CHCl_3_); IR (neat) ν_max_ 3432, 2920, 2851, 1769, 1647, 1557, 1456, 1384, 1239, 1162, 1089, 944, 874, and 776 cm^–1^; ^1^H NMR (500 MHz, CDCl_3_); and ^13^C (125 MHz, CDCl_3_) data, see Table 3. ESIMS *m/z* 383 [M + Na]^+^. HRESIMS *m/z* 383.2195 [M + Na]^+^ (calcd for C_22_H_32_O_4_Na, 383.2198).

#### 3.3.5. Preparation of (S)- and (R)-MTPA Esters of **4**

To a solution of **4a** (1 mg, 2.8 μM) in pyridine (100 *μ*L), *R*-(−)-MTPA-Cl (5 μL) was added and left to react overnight at RT. The reaction was ended by addition of water (1.0 mL), and the mixture was further processed, as previously described [53,54], to afford (*S*)-MTPA ester (**4a**, 1.4 mg, 2.4 μM). The correspondent (*R*)-MTPA ester (**4b**, 0.9 mg, 1.6 μM) was similarly obtained from the reaction of *S*-(+)-MTPA-Cl with **4**. ^1^H NMR (CDCl_3_, 400 MHz) of **4a**: δ_H_ 7.340 (1H, br dd, *J =* 1.8, 1.8 Hz, H-1), 7.208 (1H, br s, H-4), 6.275 (1H, br s, H-2), 5.164 (1H, dd, *J =* 8.0, 8.0 Hz, H-11), 5.113 (1H, m, H-7), 2.489 (1H, m, H-18a), 2.450 (2H, dd, *J =* 7.6, 7.6 Hz, H_2_-5), 2.426 (1H, m, H-18a), 1.593 (3H, H_3_-21), 1.559 (3H, H_3_-22), and 1.355 (3H, H_3_-20). ^1^H NMR (CDCl_3_, 400 MHz) of **4b**: δ_H_ 7.338 (1H, br s, H-1), 7.206 (1H, br s, H-4), 6.275 (1H, br s, H-2), 5.174 (1H, ddd, *J =* 9.2, 9.2, 3.2 Hz, H-11), 5.065 (1H, br dd, *J =* 7.8, 7.8 Hz, H-7), 2.563 (1H, m, H-18a), 2.541 (1H, m, H-18a), 2.447 (2H, dd, *J =* 8.0, 8.0 Hz, H_2_-5), 1.570 (3H, H_3_-21), 1.532 (3H, H_3_-22), and 1.376 (3H, H_3_-20).

### 3.4. In Vitro Bioassays

#### 3.4.1. Anti-Inflammatory Activity

Human neutrophils were isolated from the blood of healthy adult volunteers and enriched by using dextran sedimentation, Ficoll–Hypaque gradient centrifugation, and hypotonic lysis, as described previously [59]. Then, neutrophils were incubated in Ca^2+^-free HBSS buffer (pH 7.4, ice-cold). 

##### Superoxide Anion Generation

Neutrophils (6 × 10^5^ cells/mL) incubated (with 0.6 mg/mL ferricytochrome *c* and 1 mM Ca^2+^) in HBSS at 37 °C were treated with DMSO (as control) or tested compound for 5 min. Neutrophils were primed by 1 μg/mL cytochalasin B (CB) for 3 min before being activated by 100 nM fMLF for 10 min. The change of superoxide anion generation was spectrophotomically measured at 550 nm (U-3010, Hitachi, Tokyo, Japan) [57,58]. LY294002 [2-(4-morpholinyl)-8-phenyl-1(4*H*)-benzopyran- 4-one] was used as a positive control.

##### Elastase Release

Neutrophils (6 × 10^5^ cells/mL) incubated (with 100 μM MeO-Suc-Ala-Ala-Pro-Val-*p*- nitroanilide and 1 mM Ca^2+^) in HBSS at 37 °C were treated with DMSO or the tested compound for 5 min. Neutrophils were, then, activated with fMLF (100 nM)/CB (0.5 μg/mL) for 10 min. The change of elastase release was spectrophotomically measured at 405 nm (U-3010, Hitachi, Tokyo, Japan) [58].

#### 3.4.2. Cytotoxic Activity

P388, HuCCT-1, DLD-1, and CCD-966SK cell lines were purchased from the American Type Culture Collection (ATCC). Cytotoxicities of compounds **1**–**7** were measured using Almar Blue assay [55,56], with doxorubicin hydrochloride used as a positive control.

#### 3.4.3. Statistical Analysis

Data are displayed as the mean ± SEM and comparisons were performed by one-way ANOVA with Dunnett analysis. All results were obtained from more than 3 biological replicates. A *p* value of 0.05 or less was considered to be significant. The software Prism (GraphPad Software, San Diego, CA, USA) was used for the statistical analysis.

## 4. Conclusions

The chemical investigation of dichloromethane-soluble fraction of the organic extract of a Red Sea sponge *Spongia* sp. resulted in the isolation and identification of a rare A-ring contracted secospongian diterpenoid 17-dehydroxysponalactone (**1**) and three new furano-norterpenoids **2**–**4**. Compound **1** was found to be noncytotoxic but was shown to exhibit potent inhibitory activity against the superoxide anion generation and elastase release in the fMLF/CB-induced neutrophils, and **5** was also found to display strong inhibitory activity against the superoxide anion generation. Therefore, **1** and **5** are the promising candidates for further development of anti-inflammatory agents.

## Figures and Tables

**Table 1:** ^1^H and ^13^C NMR data (500 and 125 MHz, CDCl_3_) for **1**.

Position	δ_H_, m (*J* in Hz)	δ_C_, Type
1	3.87, 1H, br s	81.8, CH
2	-	83.3, C
3	-	180.6, C
4	-	47.6, C
5	1.90, 1H, d (11.5)	56.0, CH
6	1.63, 1H, br dd (10.5, 10.5)	18.3, CH_2_
	1.66, 1H, m	
7	1.64, 1H, m	40.0, CH_2_
	2.16, 1H, br d (10.5)	
8	-	34.4, C
9	1.96, 1H, d (11.5)	47.0, CH
10	-	46.4, C
11	1.68, 1H, m	20.2, CH_2_
	1.78, 1H, dq (12.5, 6.5)	
12	2.59, 1H, ddd (16.0, 12.5, 6.5)	19.7, CH_2_
	2.76, 1H, dd (16.0, 6.0)	
13	-	119.6, C
14	-	136.8, C
15	7.09, 1H, br s	134.8, CH
16	7.06, 1H, br s	137.1, CH
17	1.24, 3H, s	26.9, CH_3_
18	1.14, 3H, s	22.6, CH_3_
19	3.92, 1H, d (12.0)	74.6, CH_2_
	4.37, 1H, d (12.0)	
20	0.84, 3H, s	14.0, CH_3_

**Table 2:** ^1^H and ^13^C NMR data for compounds **2** and **3**.

	2		3	
#	δ_H_, m (*J* in Hz) *^a^*	δ_C_ *^b^*	δ_H_, m (*J* in Hz) *^a^*	δ_C_ *^b^*
1	7.34, 1H, brs	142.5, CH	7.35, 1H, brs	142.9, CH
2	6.27, 1H, brs	111.0, CH	6.27, 1H, brs	111.2, CH
3	-	124.7, C	-	125.1, C
4	7.20, 1H, s	138.8, CH	7.21, 1H, s	139.1, CH
5	2.45, 2H, dt (7.6, 7.6)	24.8, CH_2_	2.40, 2H, dt (7.6, 7.6)	24.4, CH_2_
6	2.25, 2H, dt (7.6, 7.2)	28.3, CH_2_	2.25, 2H, dt (7.6, 7.6)	28.1, CH_2_
7	5.22, 1H, dd (7.2, 7.2)	124.7, CH	2.08, 2H, dd (7.2, 7.6)	39.1, CH_2_
8	-	133.7, C	-	139.7, C
9	2.32, 2H, dd (7.6, 7.6)	34.2, CH_2_	5.34, 1H, dd (6.0, 6.0)	115.5, CH
10	2.47, 2H, m	32.9, CH_2_	3.10, 2H, d (6.8)	33.2, CH_2_
11	-	180.0, C	-	176.1, C
12	1.61, 3H, s	15.9, CH_3_	1.65, 3H, s	16.5, CH_3_

*^a^* Spectrum recorded at 400 MHz in CDCl_3_.*^b^* Spectrum recorded at 100 MHz in CDCl_3_.

**Table 3:** ^1^H and ^13^C NMR data for compounds **4**, **5,** and (–)-sponalisolide B.

	4			5		(‒)-Sponalisolide B	
#	δ_H_, m (*J* in Hz) *^a^*	δ_C_ *^b^*	#	δ_H_, m (*J* in Hz) *^a^*	δ_C_ *^b^*	δ_H_, m (*J* in Hz) *^c^*	δ_C_ *^d^*
1	7.34, 1H, brs	142.5, CH	1	7.34, 1H, brs	142.6, CH	7.33, 1H, t (1.6)	142.7, CH
2	6.28, 1H, brs	111.1, CH	2	6.27, 1H, brs	111.0, CH	6.26, 1H, brs	111.1, CH
3	−	124.9, C	3	−	124.7, C	−	124.9, C
4	7.21, 1H, s	138.8, CH	4	7.20, 1H, s	138.8, CH	7.20, 1H, brs	139.0, CH
5	2.45, 2H, t (7.5)	25.0, CH_2_	5	2.45, 2H, t (7.5)	24.8, CH_2_	2.44, 2H, dd (7.7, 7.3)	24.9, CH_2_
6	2.24, 2H, dt (7.5, 7.0)	28.4, CH_2_	6	2.25, 2H, dt (7.5, 7.0)	28.3, CH_2_	2.24, 2H, ddd (14.6, 7.3, 7.0)	28.5, CH_2_
7	5.16, 1H, t, (6.0)	123.9, CH	7	5.23, 1H, t (7.0)	125.1, CH	5.22, 1H, t (7.0)	125.2, CH
8	−	135.5, C	8	−	134.1, C	−	134.2, C
9	2.00, 2H, dd, (7.5, 7.0)	39.5, CH_2_	9	1.61, 3H, s	16.0, CH_3_	1.60, 3H, s	16.1, CH_3_
10	2.08, 2H, m	26.5, CH_2_	10	2.35, 2H, m	34.7, CH_2_	2.33, 2H, m	35.1, CH_2_
11	5.17, 1H, t, (6.0)	125.4, CH	11	2.34, 2H, m	34.9, CH_2_	2.33, 2H, m	34.9, CH_2_
12	−	134.3, C	12	−	173.3, C	−	173.5, C
13	2.24, 1H, m; 2.07, 1H, m	36.2, CH_2_	1’	−	175.4, C	−	175.6, C
14	1.50, 1H, m; 1.58, 1H, m	28.9, CH_2_	2’	4.50, 1H, ddd, (11.5, 8.5, 5.5)	49.3, CH	4.52, 1H, ddd, (11.7, 8.6, 5.8)	49.4, CH
15	3.51, 1H, br d (10.5)	76.7, CH	3’	2.86, 1H, ddd, (12.0, 8.5, 6.0) 2.08, 1H, qd, (11.5, 9.0)	30.7, CH_2_	2.82, 1H, ddd, (12.2, 8.6, 5.8); 2.08, 1H, qd, (11.7, 9.1)	30.7, CH_2_
16	−	88.7, C	4’	4.47, t (9.5) 4.27, 1H, ddd, (11.5, 9.5, 6.0)	66.1, CH_2_	4.45, 1H, t, (9.5); 4.27, 1H, ddd, (11.3, 9.5, 5.8)	66.2, CH_2_
17	2.63, 2H, dd (9.0, 7.5)	29.2, CH_2_	NH	6.00 brs	−	6.16 brs	−
18	1.92, 1H, ddd (13.0, 8.0, 8.0); 2.20, 1H, m	30.6, CH_2_					
19	−	176.7, C					
20	1.37, 3H, s	21.3, CH_3_					
21	1.61, 3H, s	16.0, CH_3_					
22	1.61, 3H, s	15.9, CH_3_					

*^a^* Spectrum recorded at 500 MHz in CDCl_3_.*^b^* Spectrum recorded at 125 MHz in CDCl_3_. *^c^* Spectrum recorded at 400 MHz in CDCl_3_ [42]. *^d^* Spectrum recorded at 125 MHz in CDCl_3_ [42].

**Table 4:** Selected ^13^C NMR data at C-15‒C-20 of **4** and **10** and the correspondent carbons C-7‒C-12 of the related compounds **11** and **12**.

	4 *^a^*	10 (15*R*,16*S*) *^b^*	C#	11 (7*R*,8*S*) *^c^*	12 (7*R*,8*R*) *^c^*
C-15	76.7	75.5	C-7	75.1	76.2
C-16	88.7	88.9	C-8	88.9	88.9
C-17	29.2	27.8	C-9	27.6	29.2
C-18	30.6	29.5	C-10	29.5	30.7
C-19	176.7	177.3	C-11	177.1	176.6
C-20	21.3	23.0	C-12	23.1	21.4

*^a^* Spectrum recorded at 125 MHz in CDCl_3_.*^b^* Spectrum recorded at 75 MHz in CDCl_3_ [52].*^c^* Spectrum recorded at 125 MHz in CDCl_3_ [42].

**Table 5:** Effects of compounds **1**−**7** on superoxide anion generation and elastase release in *N*-formyl-methionyl-leucyl phenylalanine/cytochalasin B (fMLF/CB)-induced human neutrophils.

Compound	Superoxide Anion								Elastase							
	IC_50_ (μM) ^a^				Inh %				IC_50_ (μM) ^a^				Inh %			
1	3.37	±	0.21		91.38	±	2.91	***	4.07	±	0.60		90.29	±	7.71	***
2		− ^b^			3.47	±	0.68	**		−			14.03	±	3.28	*
3		−			8.85	±	3.73			−			18.00	±	6.08	*
4		−			2.61	±	1.26			−			-1.07	±	7.93	
5	5.31	±	1.52		67.12	±	6.00	***		−			35.18	±	8.03	**
6		−			9.44	±	5.04			−			19.24	±	3.86	**
7		−			12.79	±	6.01			−			25.87	±	4.18	**
LY294002 ^c^	1.88	±	0.77		90.27	±	3.87	***	2.58	±	0.67		77.59	±	2.34	***

Percentage of inhibition (Inh %) at 10 μM. Results are presented as mean ± SEM (n ≥ 3). * *p* < 0.05, ** *p* < 0.01, *** *p* < 0.001 as compared with the control (DMSO). ^a^ Concentration necessary for 50% inhibition (IC_50_). ^b^ The compound is not considered to be anti-inflammatory when IC_50_ value is >10 μM. ^c^ A phosphatidylinositol-3-kinase inhibitor was used as a positive control.

## Data Availability

Data available in a publicly accessible repository.

## Supplementary Materials

HRESIMS, ^1^H, ^13^C, DEPT, HMQC, COSY, HMBC ,and NOESY spectra of new compounds **1**–**4** are available online at https://www.mdpi.com/1660-3397/19/1/38/s1, Figure S1: HRESIMS spectrum of **1**, Figure S2: ^1^H NMR spectrum of **1** in CDCl_3_ at 500 MHz, Figure S3: ^13^C NMR spectrum of **1** in CDCl_3_ at 125 MHz, Figure S4: HSQC spectrum of **1** in CDCl_3_, Figure S5: ^1^H -^1^H COSY spectrum of **1** in CDCl_3_, Figure S6: HMBC spectrum of **1** in CDCl_3_, Figure S7: NOESY spectrum of **1** in CDCl_3_, Figure S8: HRESIMS spectrum of **2**, Figure S9: ^1^H NMR spectrum of **2** in CDCl_3_ at 400 MHz, Figure S10: ^13^C NMR spectrum of **2** in CDCl_3_ at 100 MHz, Figure S11: HSQC spectrum of **2** in CDCl_3_, Figure S12: ^1^H -^1^H COSY spectrum of **2** in CDCl_3_, Figure S13: HMBC spectrum of **2** in CDCl_3_, Figure S14: NOESY spectrum of **2** in CDCl_3_, Figure S15: HRESIMS spectrum of **3**, Figure S16: ^1^H NMR spectrum of **3** in CDCl_3_ at 400 MHz, Figure S17: ^13^C NMR spectrum of **3** in CDCl_3_ at 100 MHz, Figure S18: HSQC spectrum of **3** in CDCl_3_, Figure S19: ^1^H -^1^H COSY spectrum of **3** in CDCl_3_, Figure S20: HMBC spectrum of **3** in CDCl_3_, Figure S21: NOESY spectrum of **3** in CDCl_3_, Figure S22: HRESIMS spectrum of **4**, Figure S23: ^1^H NMR spectrum of **4** in CDCl_3_ at 500 MHz, Figure S24: ^13^C NMR spectrum of **4** in CDCl_3_ at 125 MHz, Figure S25: HSQC spectrum of **4** in CDCl_3_, Figure S26: ^1^H NMR spectrum of **4** in CD_3_OD at 400 MHz, figure S27: ^13^C NMR spectrum of **4** in CD_3_OD at 100 MHz, Figure S28: HSQC spectrum of **4** in CD_3_OD, Figure S29: ^1^H -^1^H COSY spectrum of **4** in CD_3_OD, Figure S30: HMBC spectrum of **4** in CD_3_OD, Figure S31: NOESY spectrum of **4** in CD_3_OD, Figure S32: HRESIMS spectrum of **5**, Figure S33: ^1^H NMR spectrum of **5** in CDCl_3_ at 500 MHz, Figure S34: ^13^C NMR spectrum of **5** in CDCl_3_ at 125 MHz, Figure S35: HSQC spectrum of **5** in CDCl_3_.
