# Supplementary material for: An Anti-Inflammatory 2,4-Cyclized-3,4-Secospongian Diterpenoid and Furanoterpene-Related Metabolites of a Marine Sponge Spongia sp. from the Red Sea
Source: Mar Drugs. 2021 Jan 16;19(1):38. doi: 10.3390/md19010038 (PMC7830757; doi:10.3390/md19010038)
Supplement: Supplementary file 1 [file marinedrugs-19-00038-s001.pdf]

An Anti-inflammatory 2,4-Cyclized-3,4-Secospongian Diterpenoid and Furanoterpene-related Metabolites of a Marine Sponge *Spongia* sp. from the Red Sea

Chi-Jen Tai [1], Chiung-Yao Huang [2], Atallah F. Ahmed [3,4,*], Raha S. Orfali [3], Walied M. Alarif [5], Yusheng M. Huang [6,7], Yi-Hsuan Wang [8], Tsong-Long Hwang [8,9,10], Jyh-Horng Sheu [1,2,11,12*]

[1] Doctoral Degree Program in Marine Biotechnology, National Sun Yat-sen University, Kaohsiung 80424, Taiwan; chijentai@g-mail.nsysu.edu.tw
[2] Department of Marine Biotechnology and Resources, National Sun Yat-sen University, Kaohsiung 80424, Taiwan; betty8575@yahoo.com.tw
[3] Department of Pharmacognosy, College of Pharmacy, King Saud University, Riyadh 11451, Saudi Arabia; rorfali@ksu.edu.sa
[4] Department of Pharmacognosy, Faculty of Pharmacy, Mansoura University, Mansoura 35516, Egypt
[5] Department of Marine Chemistry, Faculty of Marine Sciences, King Abdulaziz University, Jeddah 21589, Saudi Arabia; welaref@kau.edu.sa
[6] Department of Marine Recreation, National Penghu University of Science and Technology, Magong, Penghu 88046, Taiwan; yusheng@gms.npu.edu.tw
[7] Tropical Island Sustainable Development Research Center, National Penghu University of Science and Technology, Magong, Penghu 88046, Taiwan
[8] Graduate Institute of Natural Products, College of Medicine, Chang Gung University, Taoyuan 33302, Taiwan; d0901501@cgu.edu.tw (Y.-H.W.), htl@mail.cgu.edu.tw (T.-L.H.)
[9] Research Center for Chinese Herbal Medicine, Research Center for Food and Cosmetic Safety, Graduate In-stitute of Health Industry Technology, College of Human Ecology, Chang Gung University of Science and Technology, Taoyuan, 33303, Taiwan
[10] Department of Anesthesiology, Chang Gung Memorial Hospital, Taoyuan 33305, Taiwan
[11] Graduate Institute of Natural Products, Kaohsiung Medical University, Kaohsiung 80708, Taiwan
[12] Department of Medical Research, China Medical University Hospital, China Medical University, Taichung 404333, Taiwan
* Correspondence: afahmed@ksu.edu.sa (A.F.A), Tel: +966-114-677264, Fax: +966-114-677245; sheu@mail.nsysu.edu.tw (J.-H.S.), Tel.: +886-7-525-2000 (ext. 5030); Fax: +886-7-525-5020

Table of Contents

No.	Content	Page
**Figure S1.**	HRESIMS spectrum of **1**.	**1**
**Figure S2.**	^{1}H NMR spectrum of **1** in $CDCl_3$ at 500 MHz.	**2**
**Figure S3.**	^{13}C NMR spectrum of **1** in $CDCl_3$ at 125 MHz.	**3**
**Figure S4.**	HSQC spectrum of **1** in $CDCl_3$.	**4**
**Figure S5.**	^{1}H -^{1}H COSY spectrum of **1** in $CDCl_3$.	**5**
**Figure S6.**	HMBC spectrum of **1** in $CDCl_3$.	**6**
**Figure S7.**	NOESY spectrum of **1** in $CDCl_3$.	**7**
**Figure S8.**	HRESIMS spectrum of **2**.	**8**
**Figure S9.**	^{1}H NMR spectrum of **2** in $CDCl_3$ at 400 MHz.	**9**
**Figure S10.**	^{13}C NMR spectrum of **2** in $CDCl_3$ at 100 MHz.	**10**
**Figure S11.**	HSQC spectrum of **2** in $CDCl_3$.	**11**
**Figure S12.**	^{1}H -^{1}H COSY spectrum of **2** in $CDCl_3$.	**12**
**Figure S13.**	HMBC spectrum of **2** in $CDCl_3$.	**13**
**Figure S14.**	NOESY spectrum of **2** in $CDCl_3$.	**14**
**Figure S15.**	HRESIMS spectrum of **3**.	**15**
**Figure S16.**	^{1}H NMR spectrum of **3** in $CDCl_3$ at 400 MHz.	**16**
**Figure S17.**	^{13}C NMR spectrum of **3** in $CDCl_3$ at 100 MHz.	**17**
**Figure S18.**	HSQC spectrum of **3** in $CDCl_3$.	**18**
**Figure S19.**	^{1}H -^{1}H COSY spectrum of **3** in $CDCl_3$.	**19**
**Figure S20.**	HMBC spectrum of **3** in $CDCl_3$.	**20**
**Figure S21.**	NOESY spectrum of **3** in $CDCl_3$.	**21**
**Figure S22.**	HRESIMS spectrum of **4**.	**22**

Figure S23. ^{1}H NMR spectrum of **4** in $CDCl_3$ at 500 MHz. **23**

Figure S24. ^{13}C NMR spectrum of **4** in $CDCl_3$ at 125 MHz. **24**

Figure S25. HSQC spectrum of **4** in $CDCl_3$. **25**

Figure S26. ^{1}H NMR spectrum of **4** in CD_3OD at 400 MHz. **26**

Figure S27. ^{13}C NMR spectrum of **4** in CD_3OD at 100 MHz. **27**

Figure S28. HSQC spectrum of **4** in CD_3OD. **28**

Figure S29. ^{1}H -^{1}H COSY spectrum of **4** in CD_3OD. **29**

Figure S30. HMBC spectrum of **4** in CD_3OD. **30**

Figure S31. NOESY spectrum of **4** in CD_3OD. **31**

Figure S32. HRESIMS spectrum of **5**. **32**

Figure S33. ^{1}H NMR spectrum of **5** in $CDCl_3$ at 500 MHz. **33**

Figure S34. ^{13}C NMR spectrum of **5** in $CDCl_3$ at 125 MHz. **34**

Figure S35. HSQC spectrum of **5** in $CDCl_3$. **35**

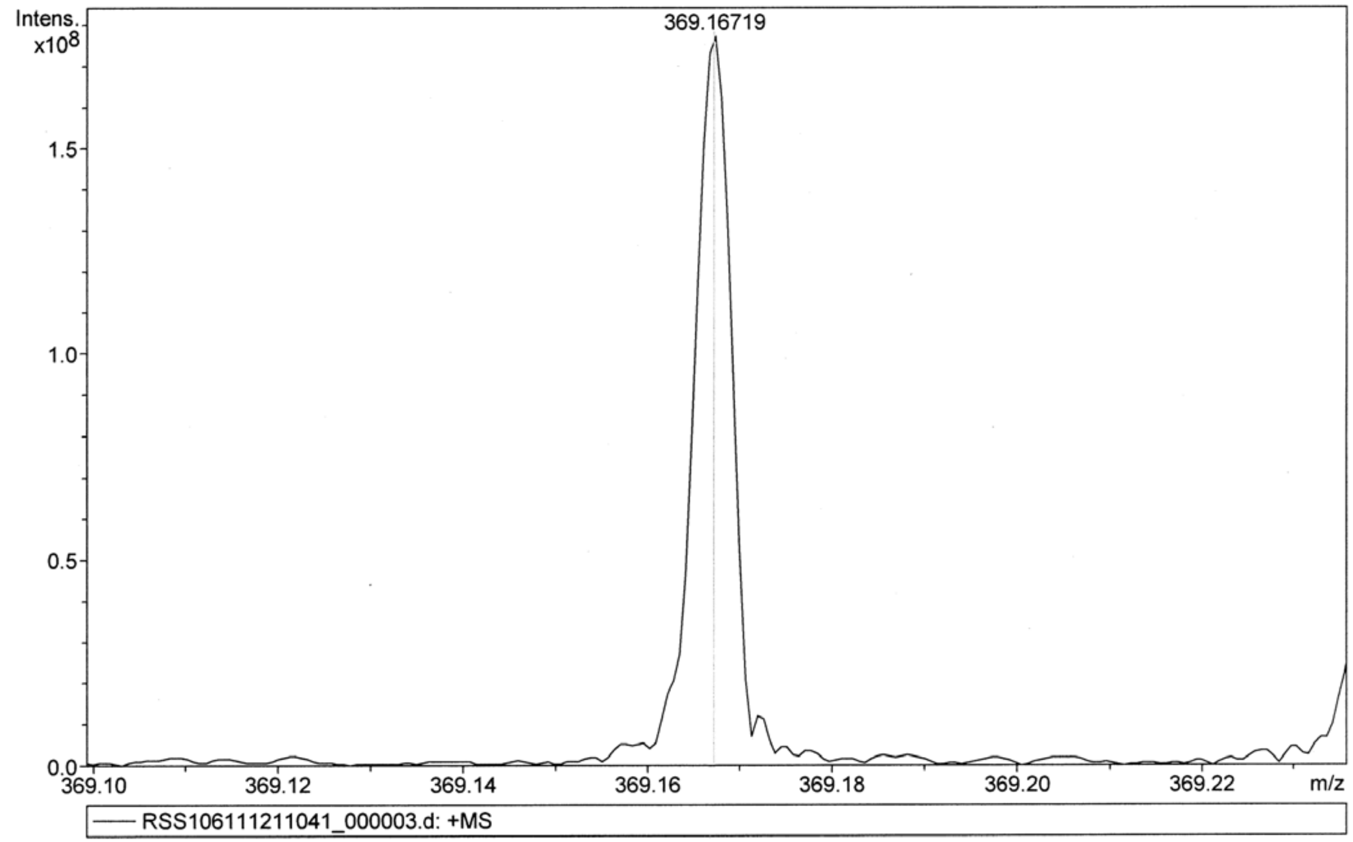

Meas. m/z	#	Formula	Score	m/z	err [mDa]	err [ppm]	mSigma	rdb	e¯ Conf	N-Rule
369.16719	1	C 20 H 26 Na O 5	100.00	369.16725	0.06	0.15	18.8	7.5	even	ok

Figure S1. HRESIMS spectrum of compound **1**.

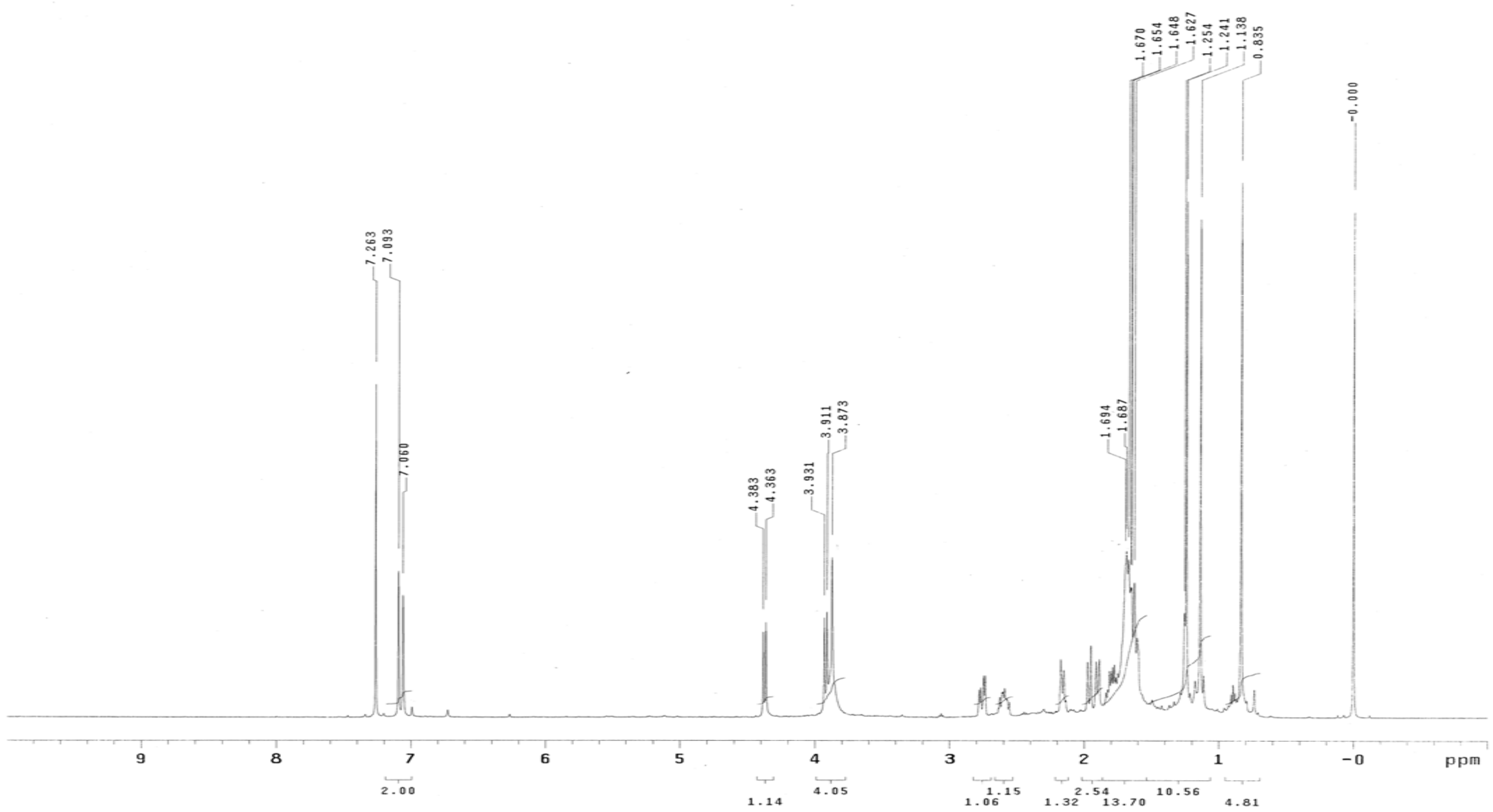

Figure S2. ^{1}H NMR spectrum (500 MHz) of compound **1** in $CDCl_3$.

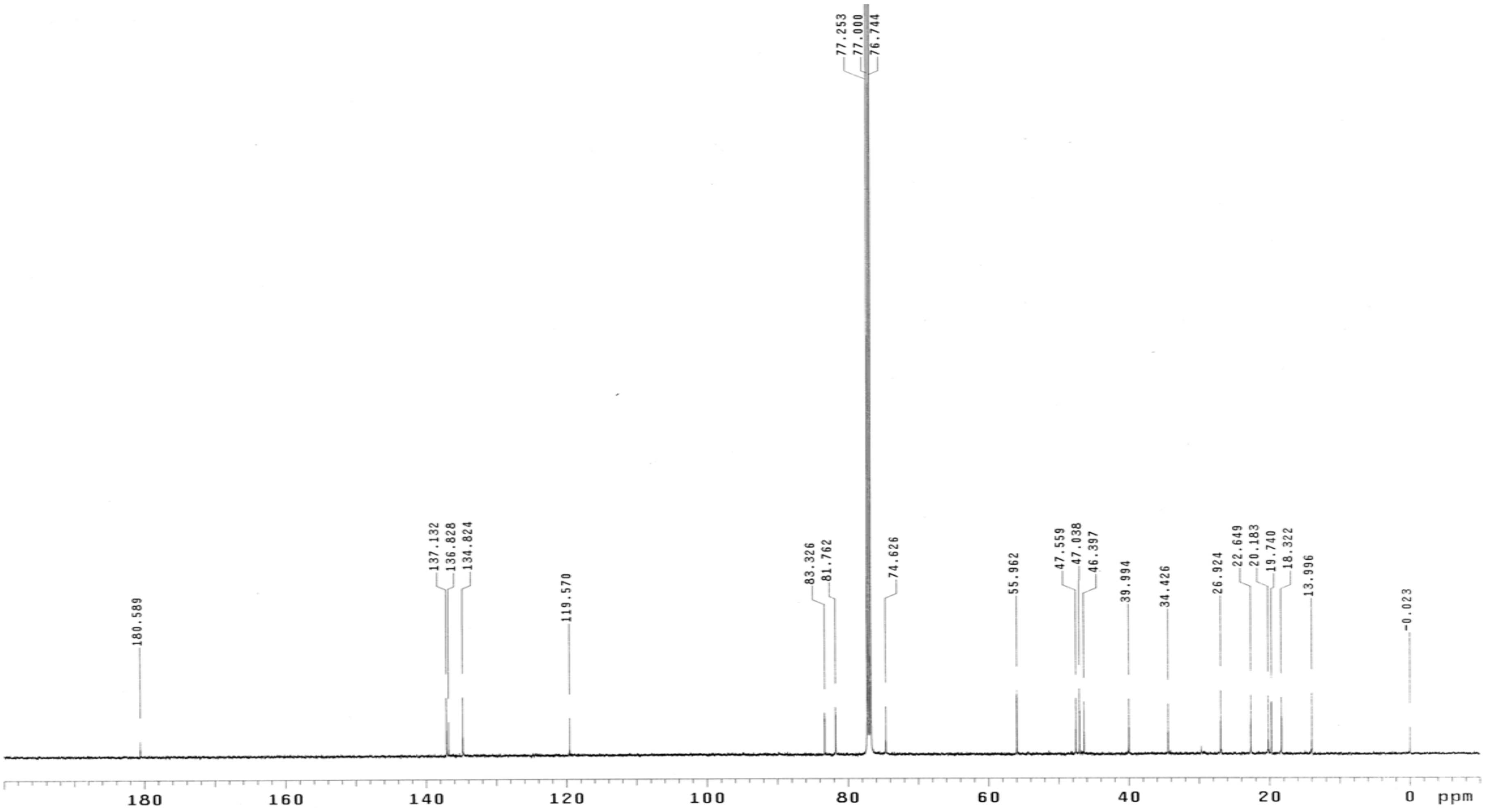

Figure S3. ^{13}C NMR spectrum (125 MHz) of compound **1** in $CDCl_3$.

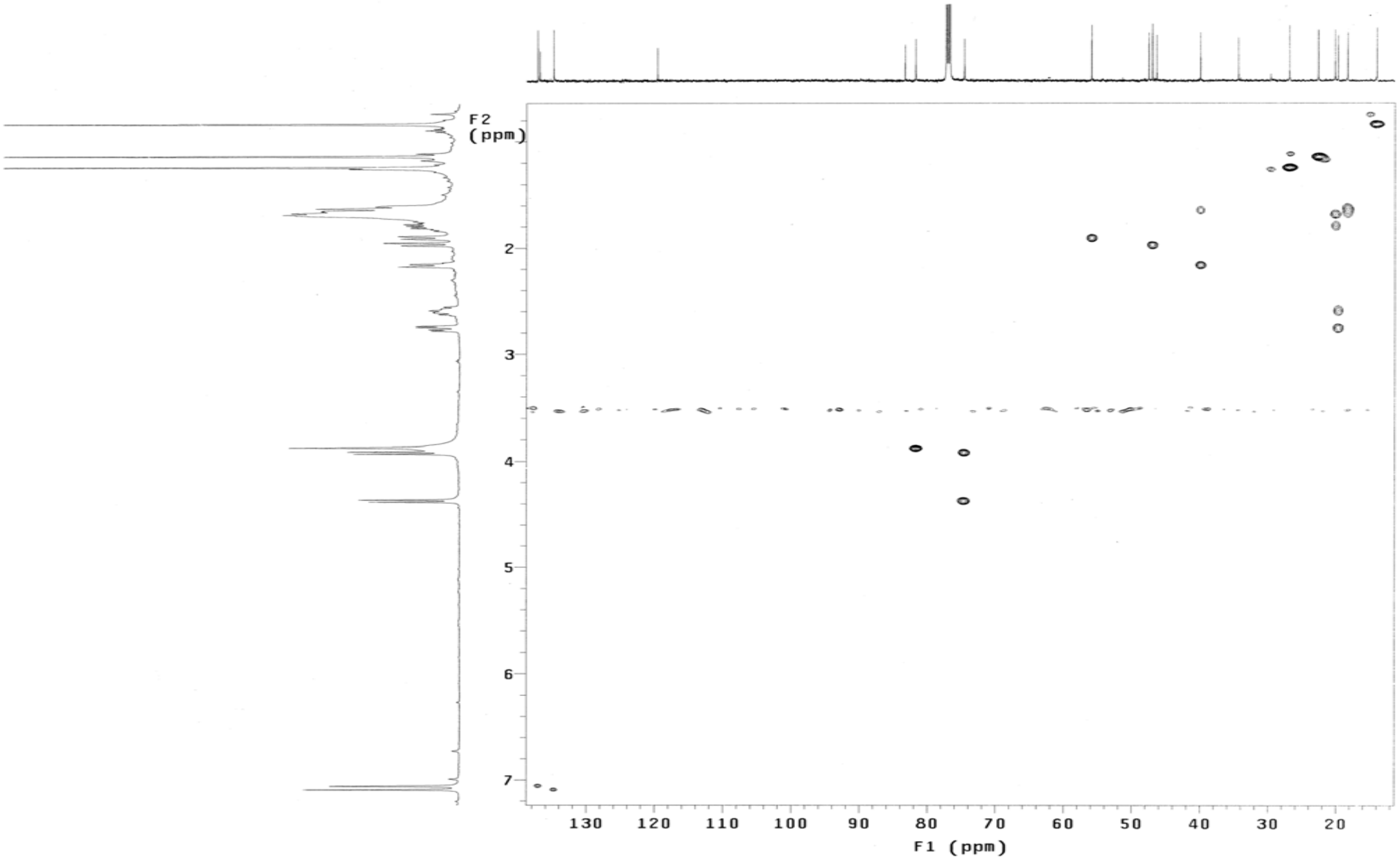

Figure S4. HSQC spectrum of **1** in $CDCl_3$.

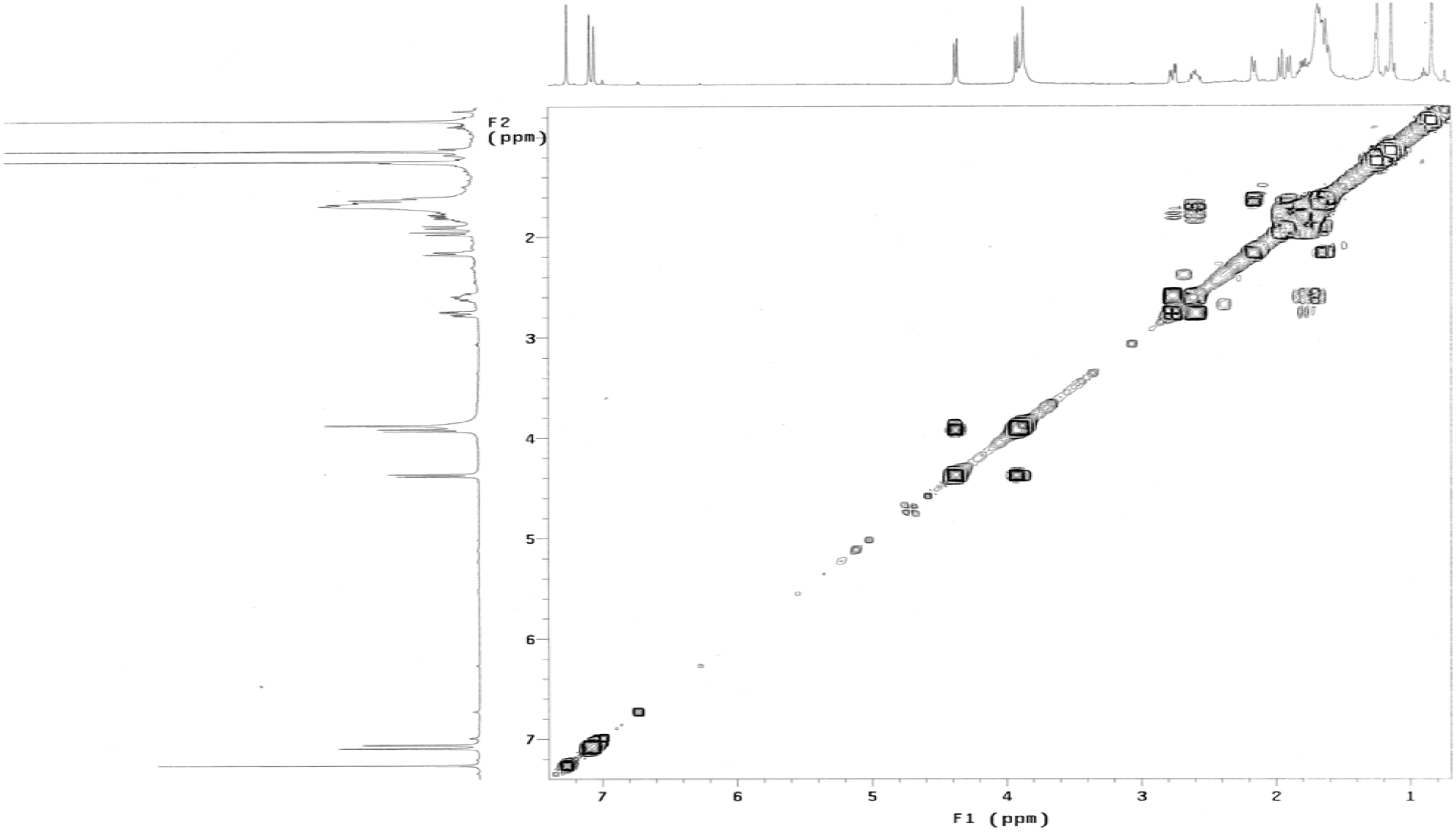

Figure S5. ^{1}H -^{1}H COSY spectrum of **1** in $CDCl_3$.

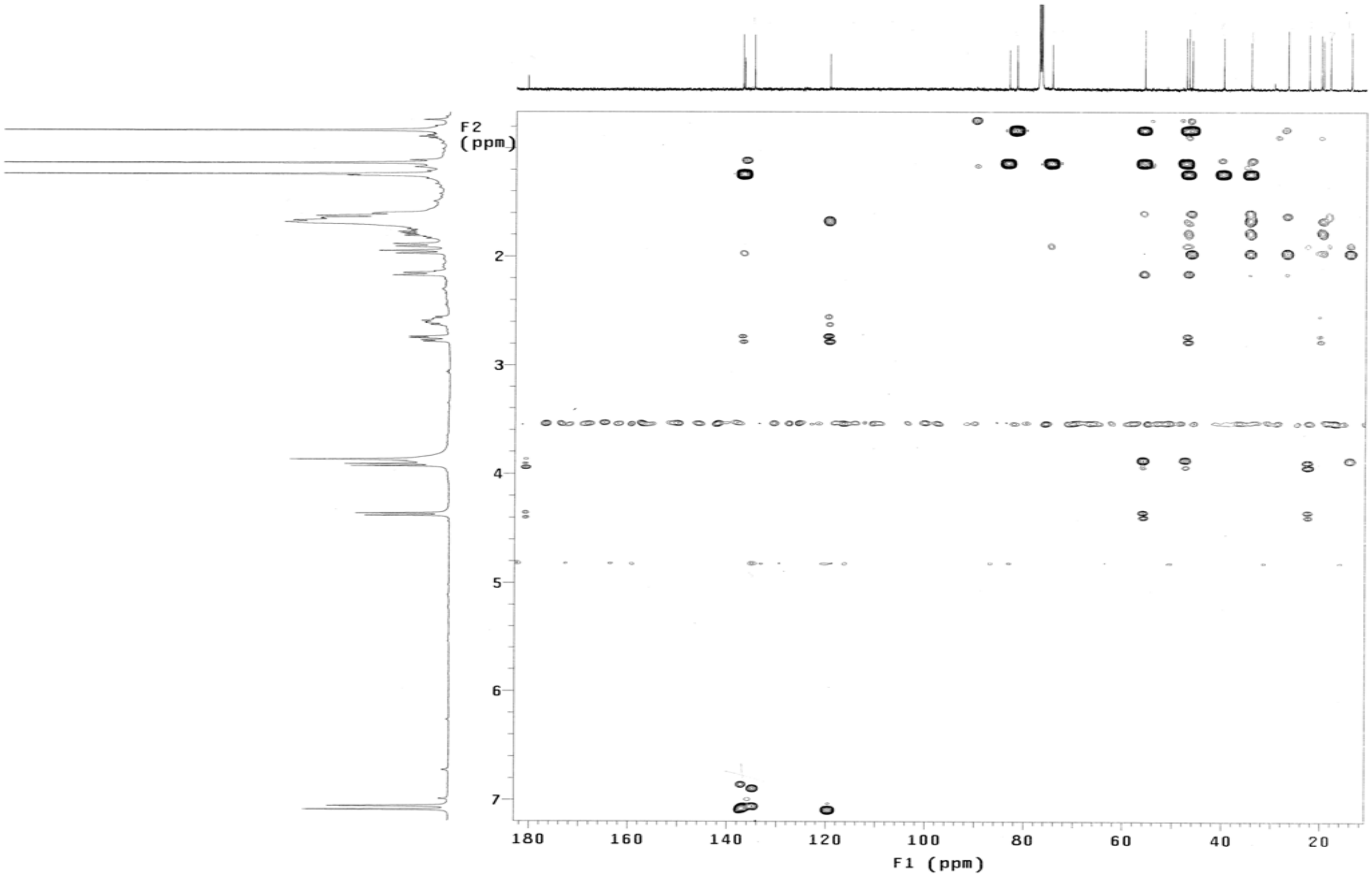

Figure S6. HMBC spectrum of **1** in $CDCl_3$.

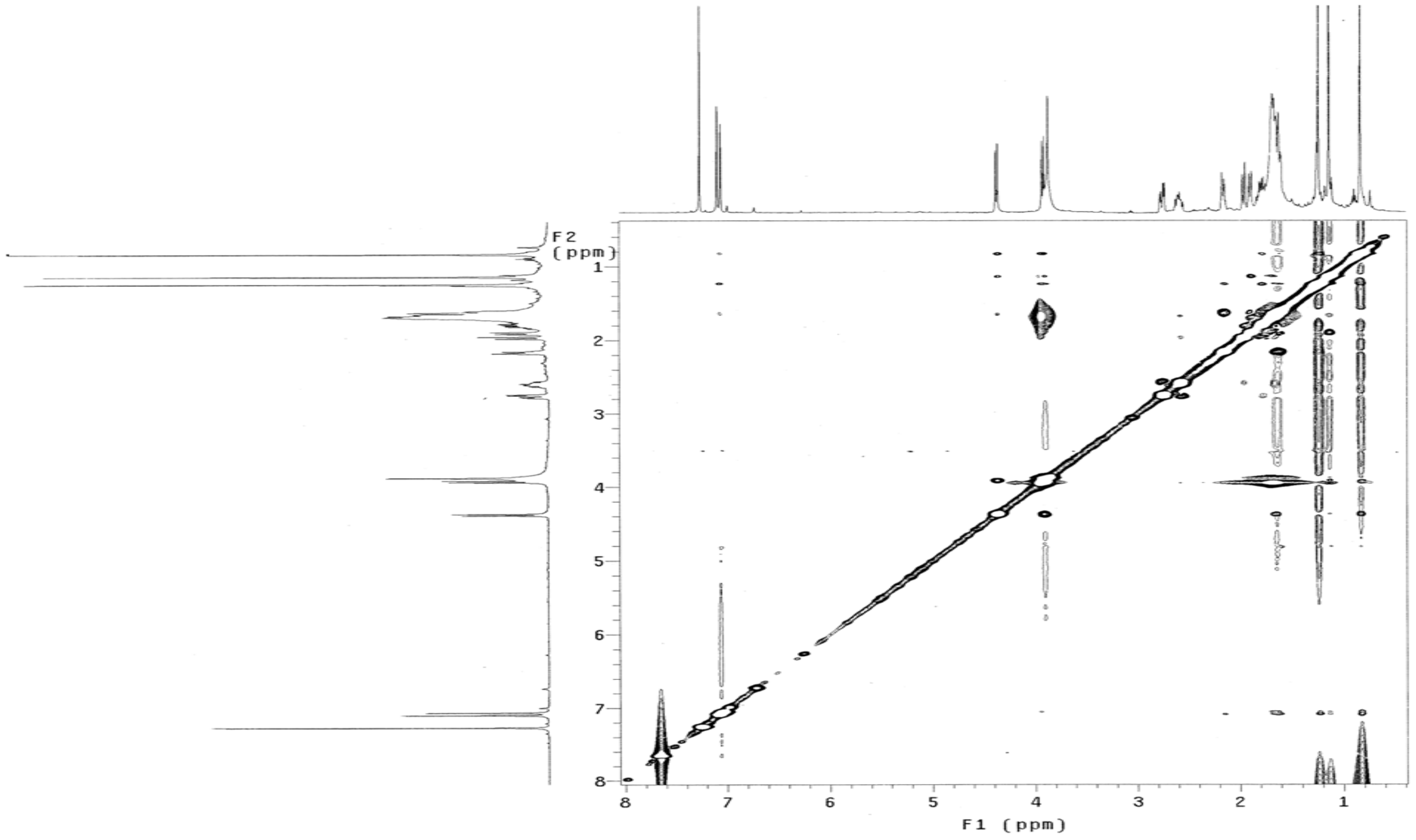

Figure S7. NOESY spectrum of **1** in $CDCl_3$.

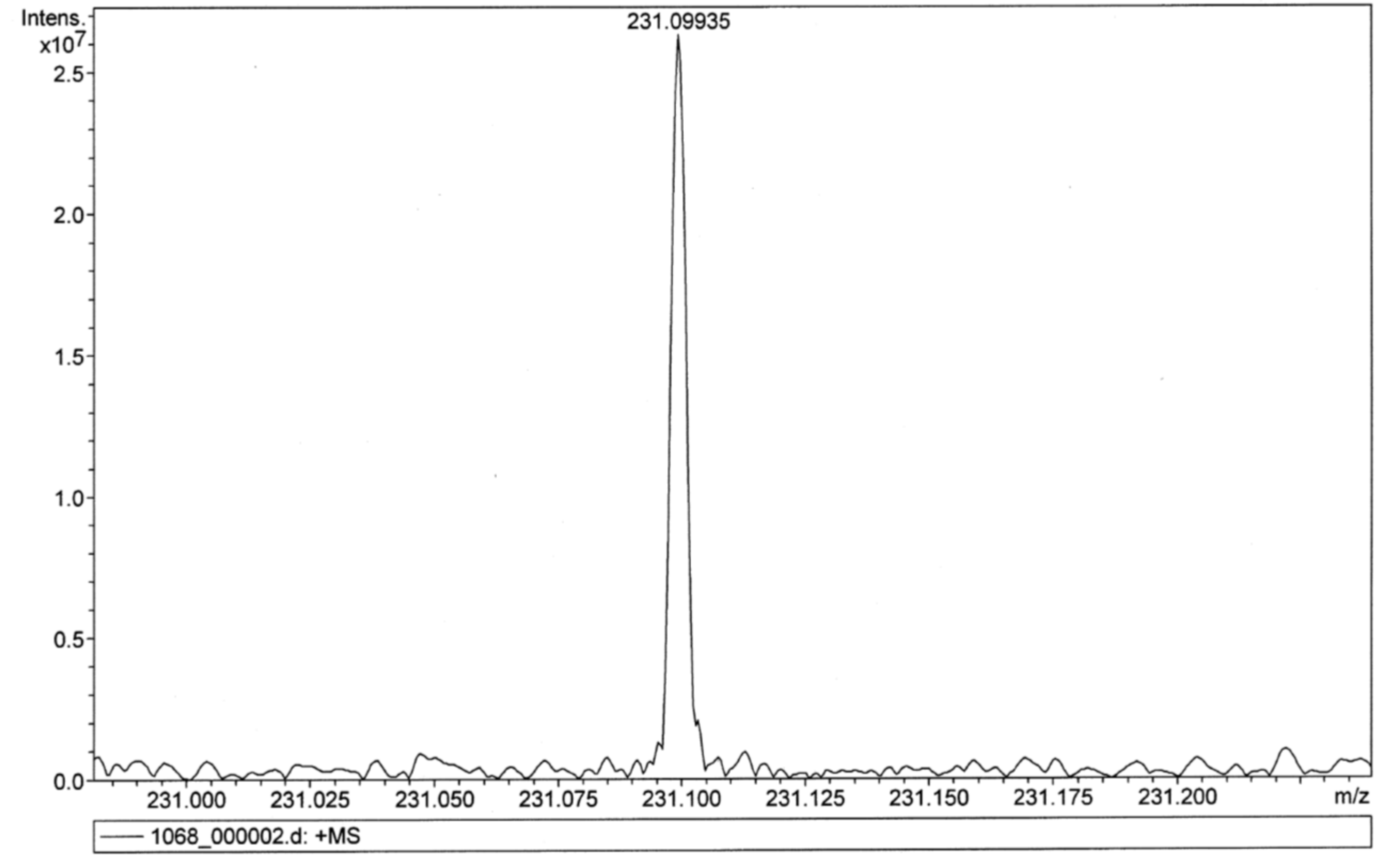

Meas. m/z	#	Formula	Score	m/z	err [mDa]	err [ppm]	mSigma	rdb	e⁻ Conf	N-Rule
231.09935	1	C 12 H 16 Na O 3	100.00	231.09917	-0.18	-0.80	9.1	4.5	even	ok

Figure S8. HRESIMS spectrum of **2**.

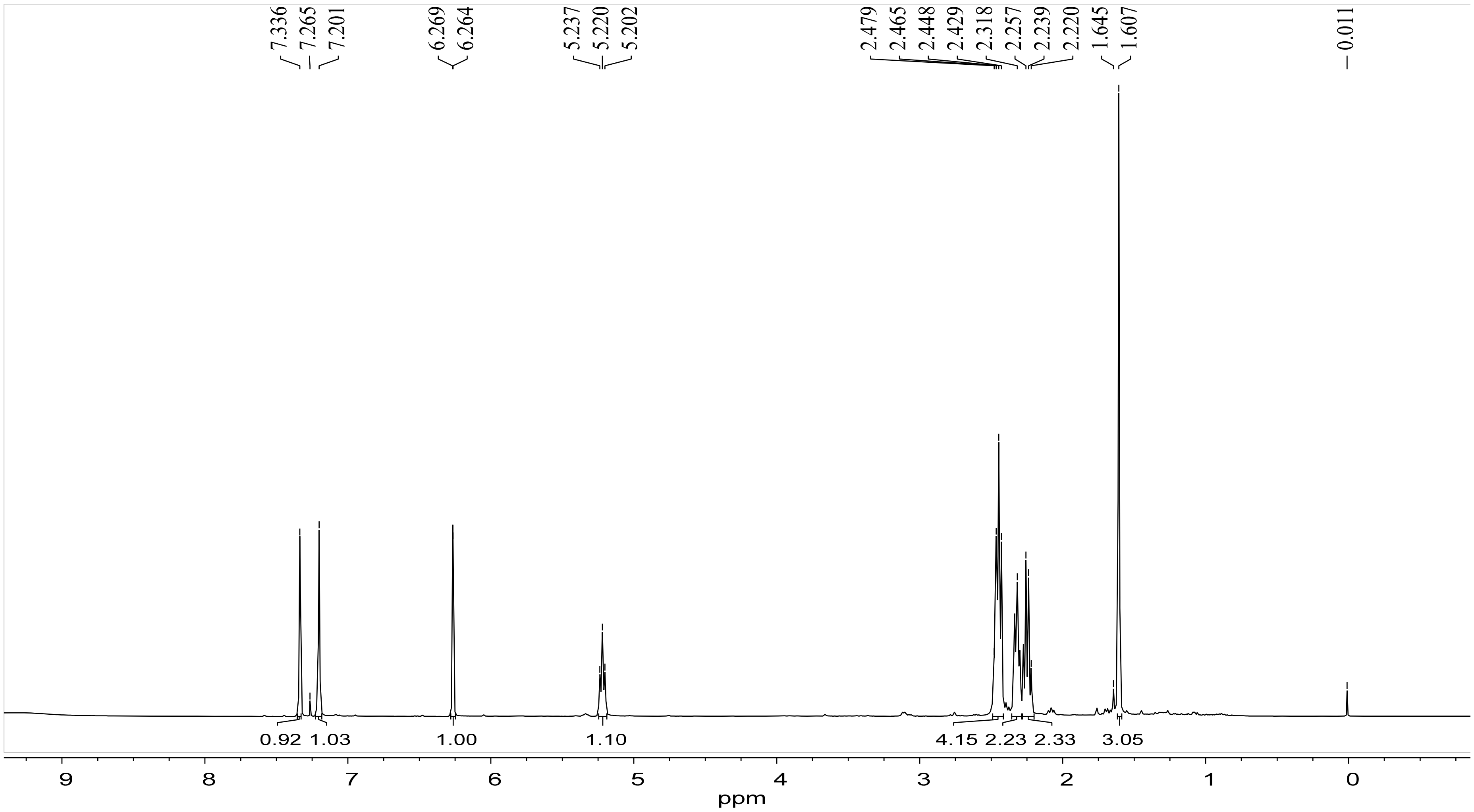

Figure S9. ^{1}H NMR spectrum (400 MHz) of compound **2** in $CDCl_3$.

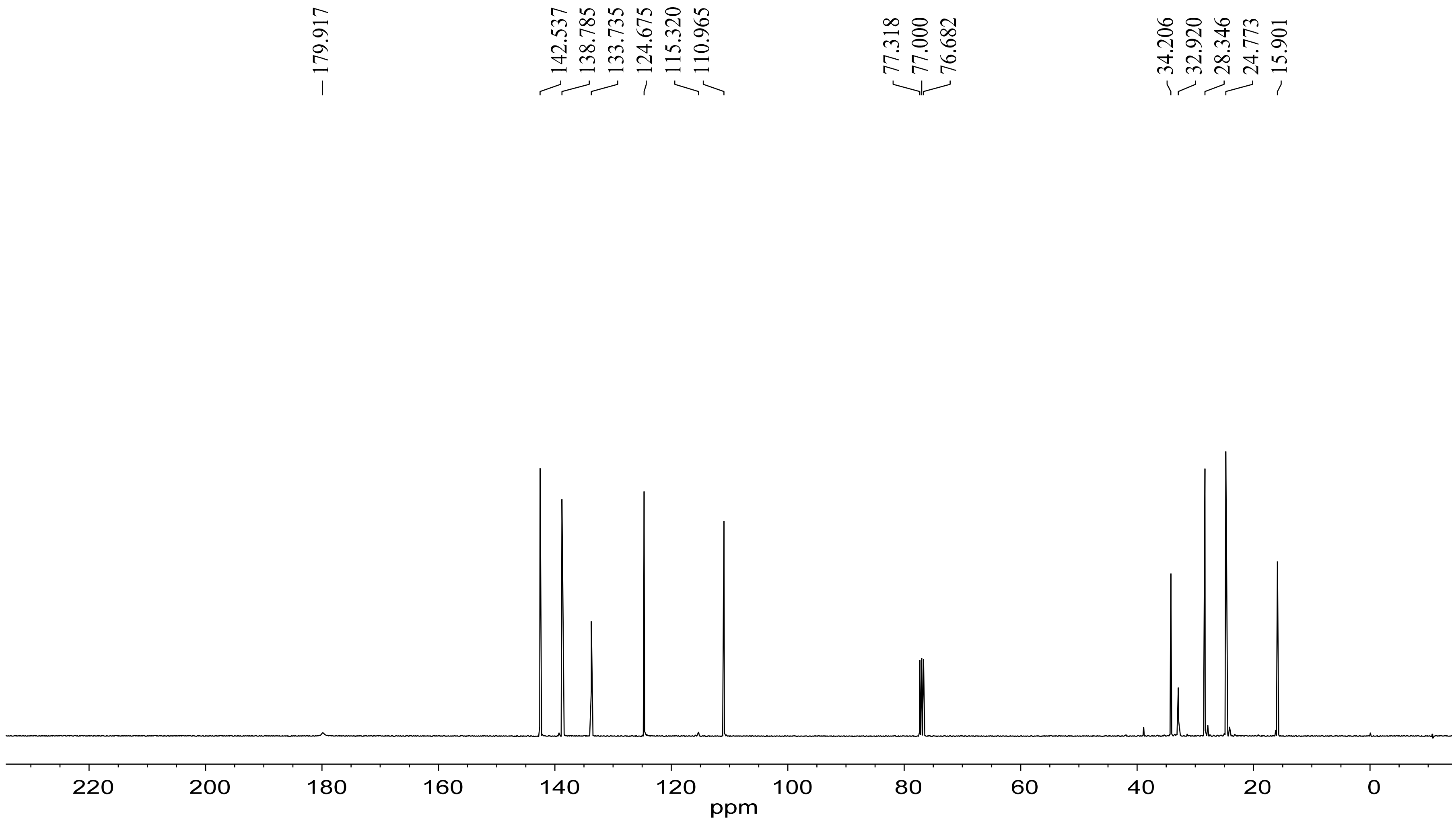

Figure S10. ^{13}C NMR spectrum (100 MHz) of compound **2** in $CDCl_3$.

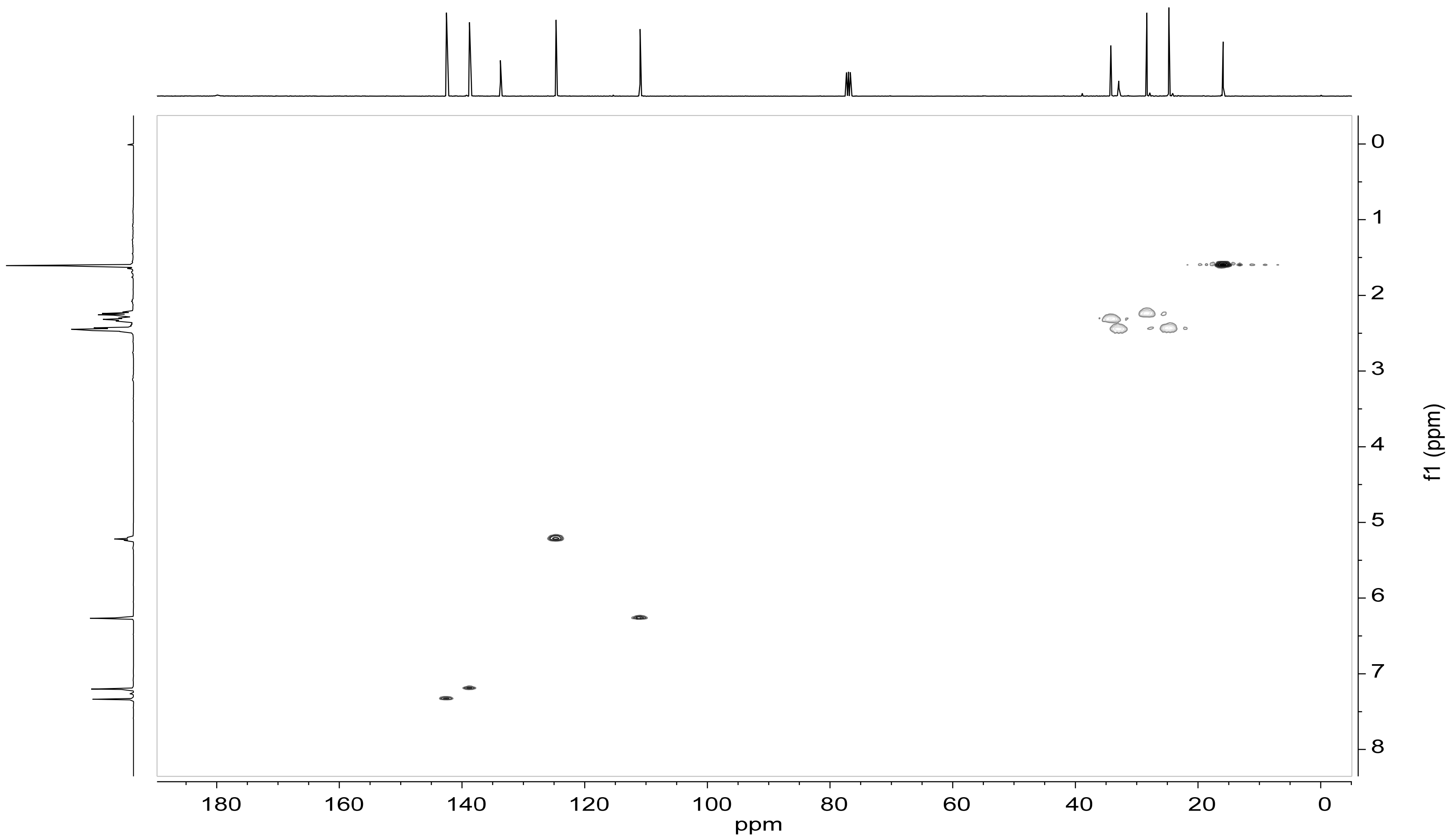

Figure S11. HSQC spectrum of **2** in $CDCl_3$.

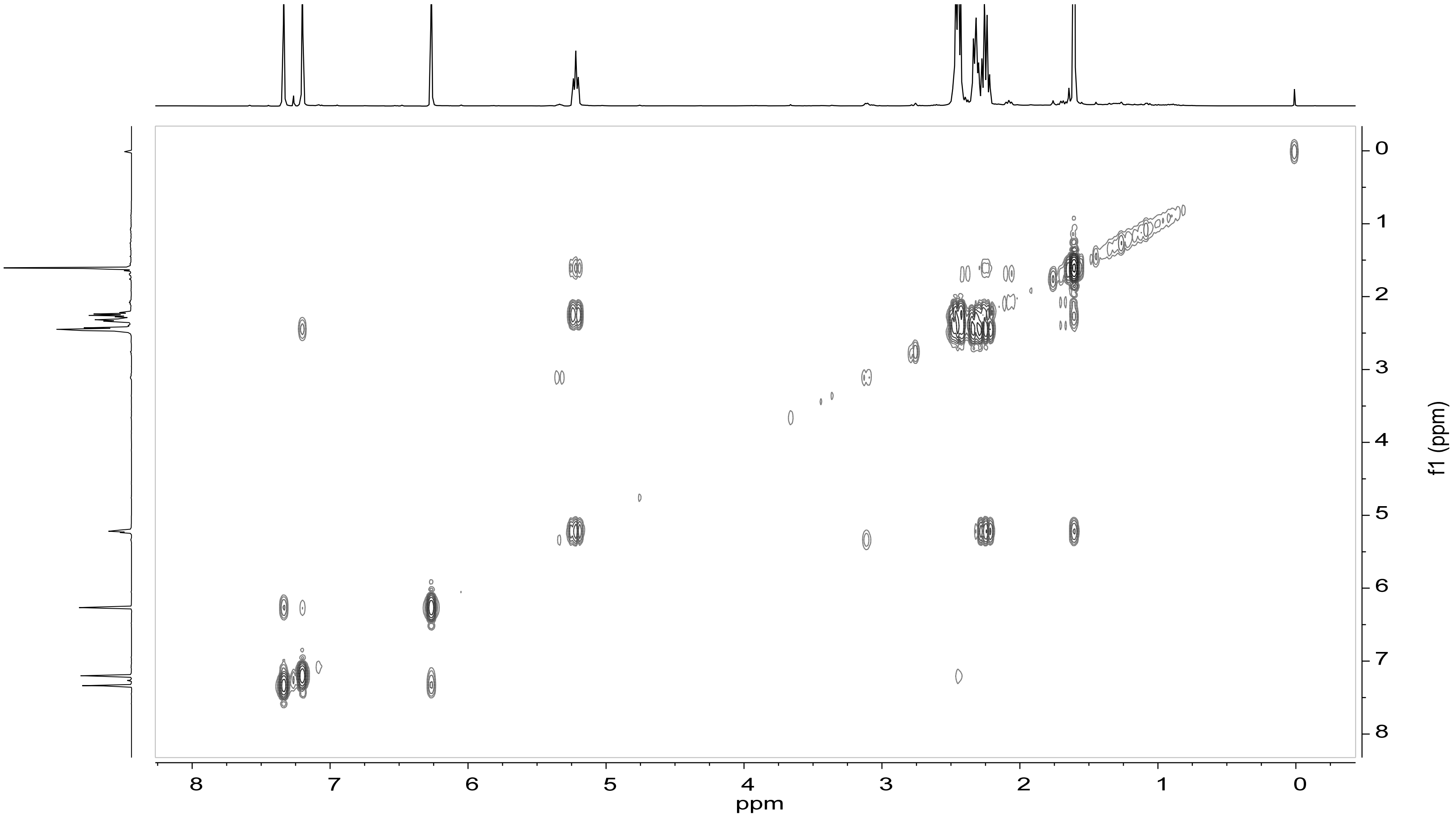

Figure S12. ^{1}H -^{1}H COSY spectrum of **2** in $CDCl_3$.

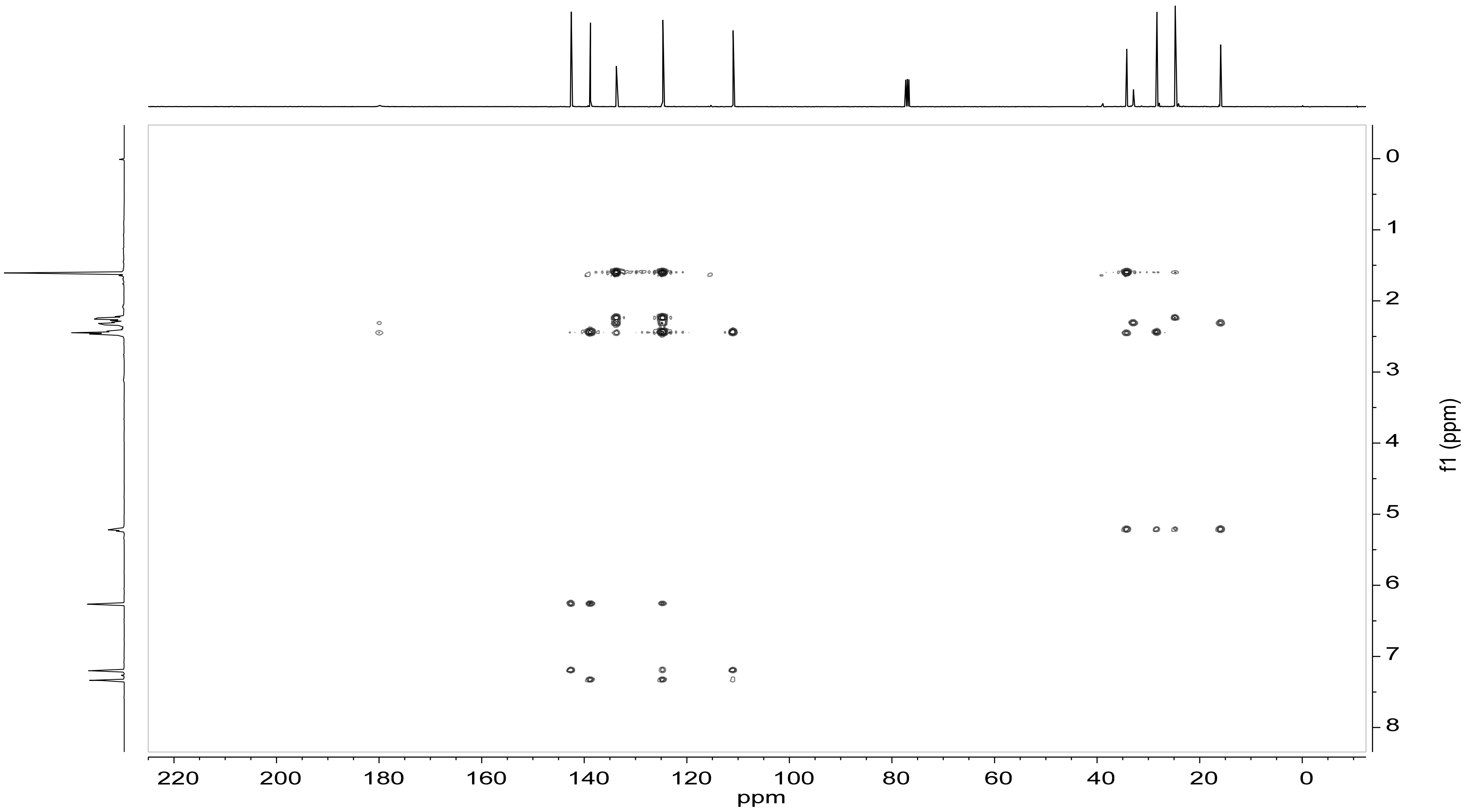

Figure S13. HMBC spectrum of **2** in $CDCl_3$.

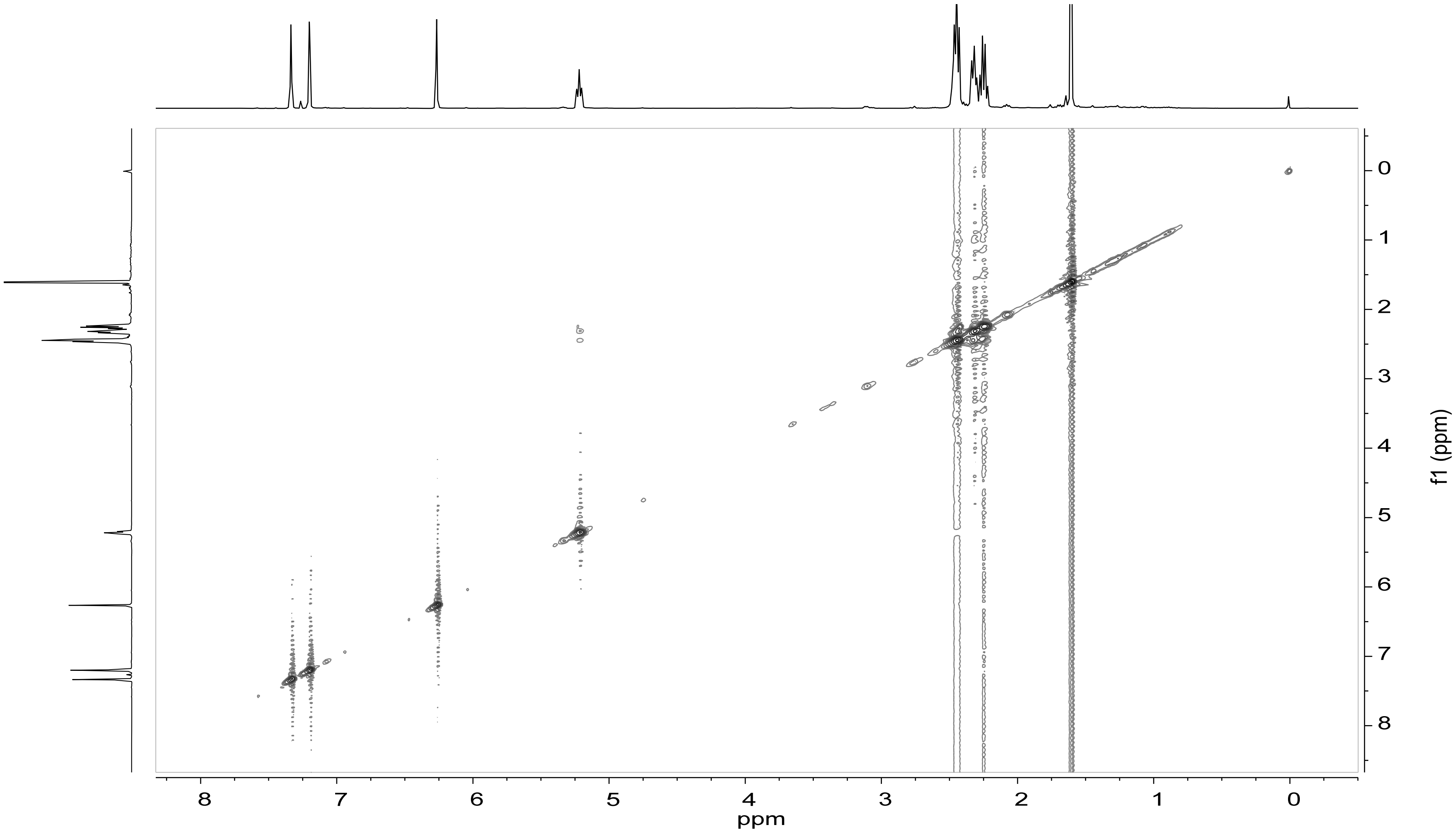

Figure S14. NOESY spectrum of **2** in $CDCl_3$.

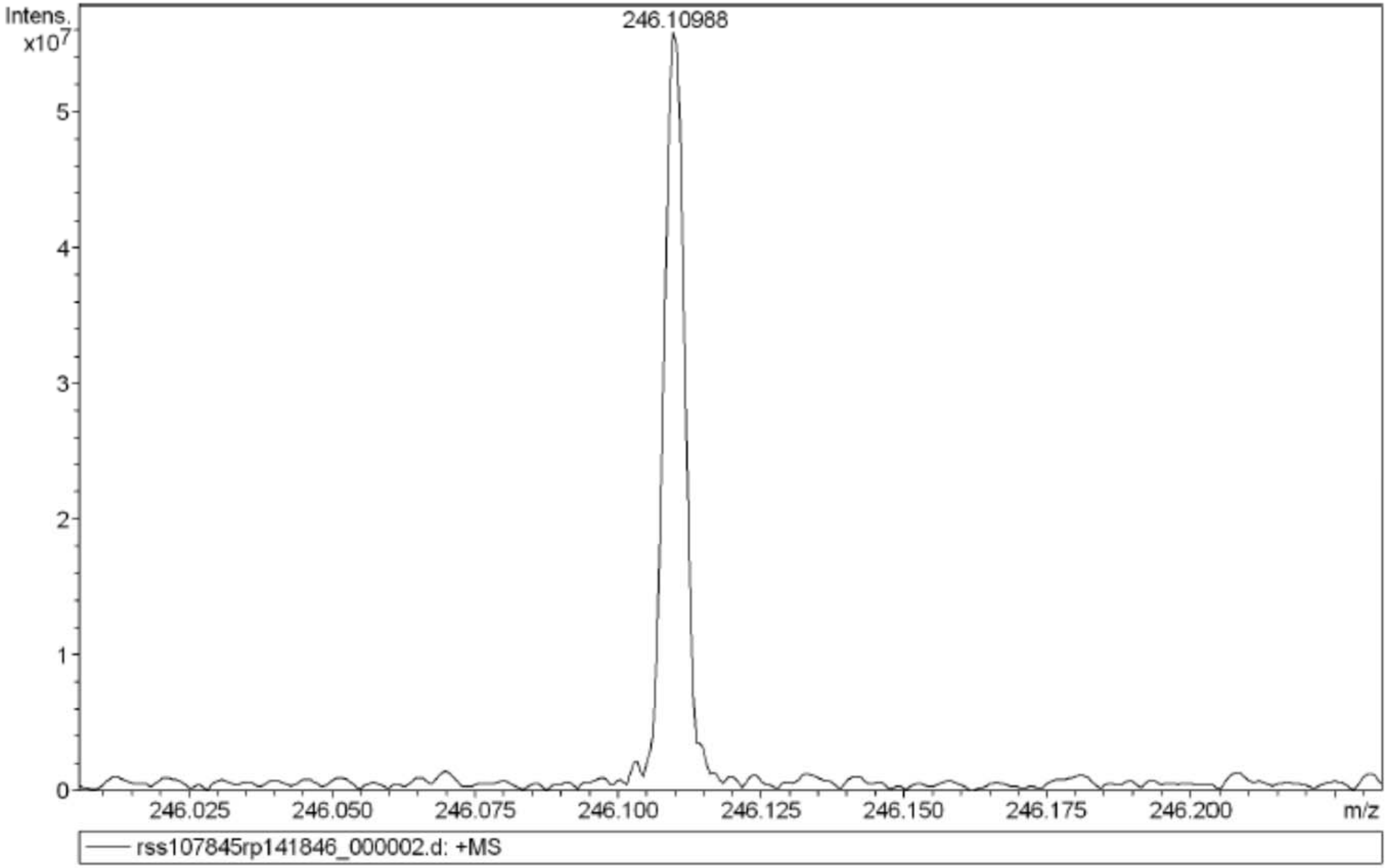

Meas. m/z	#	Formula	Score	m/z	err [mDa]	err [ppm]	mSigma	rdb	e¯ Conf	N-Rule
246.10988	1	C 12 H 17 N Na O 3	100.00	246.11006	0.18	0.74	12.1	4.5	even	ok

Figure S15. HRESIMS spectrum of compound **3**.

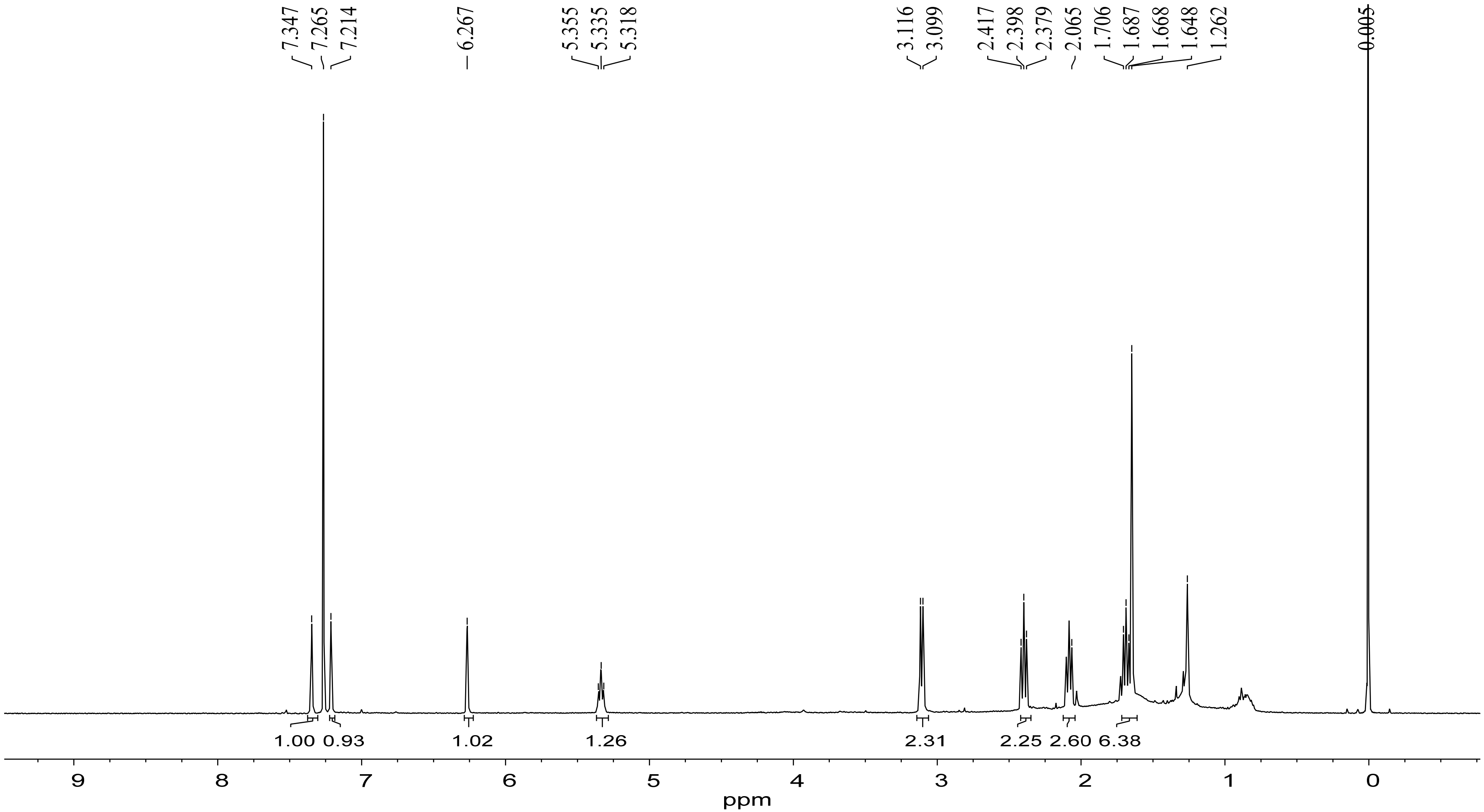

Figure S16. ^{1}H NMR spectrum (400 MHz) of compound **3** in $CDCl_3$.

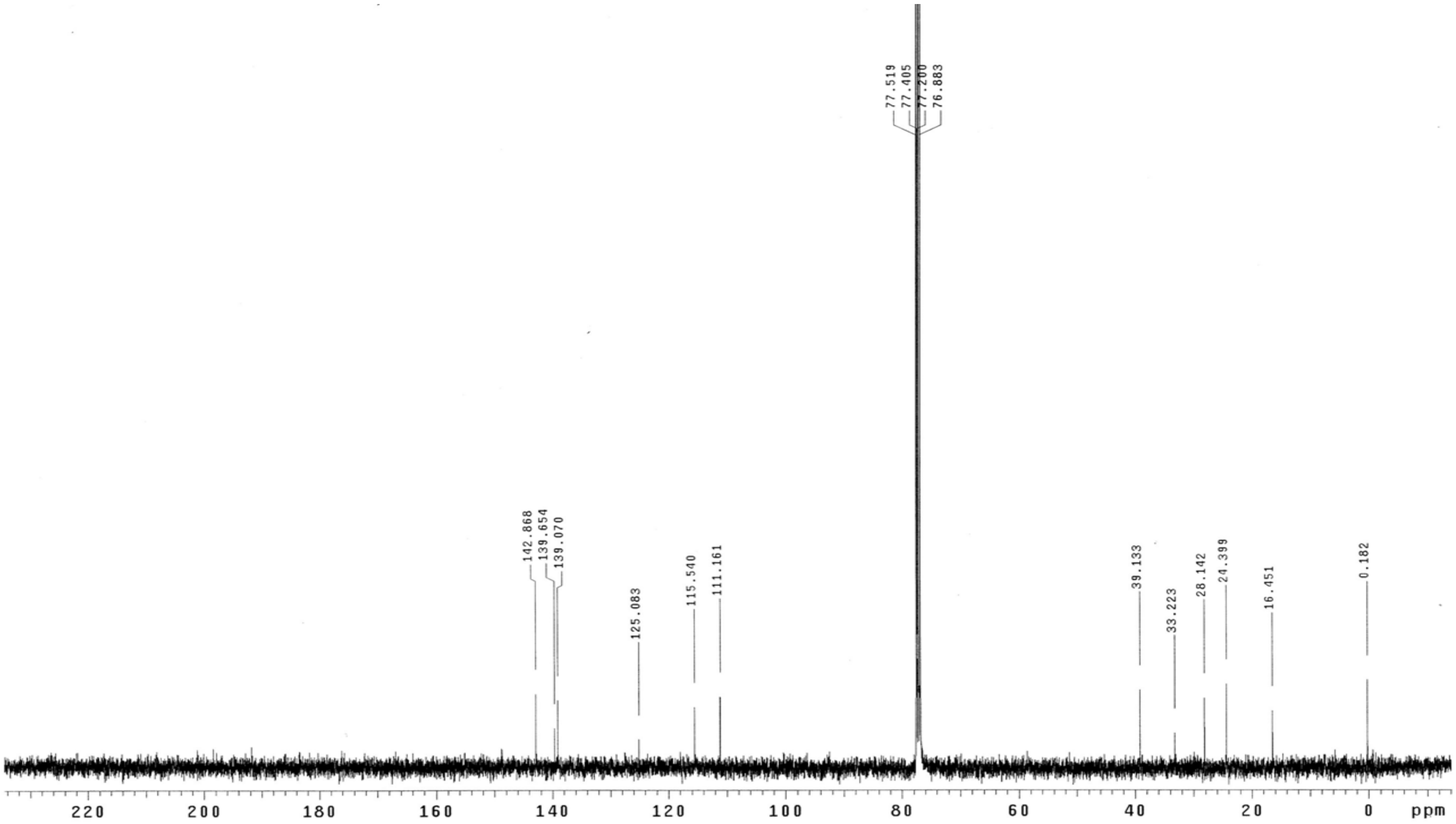

Figure S17. ^{13}C NMR spectrum (100 MHz) of compound **3** in $CDCl_3$.

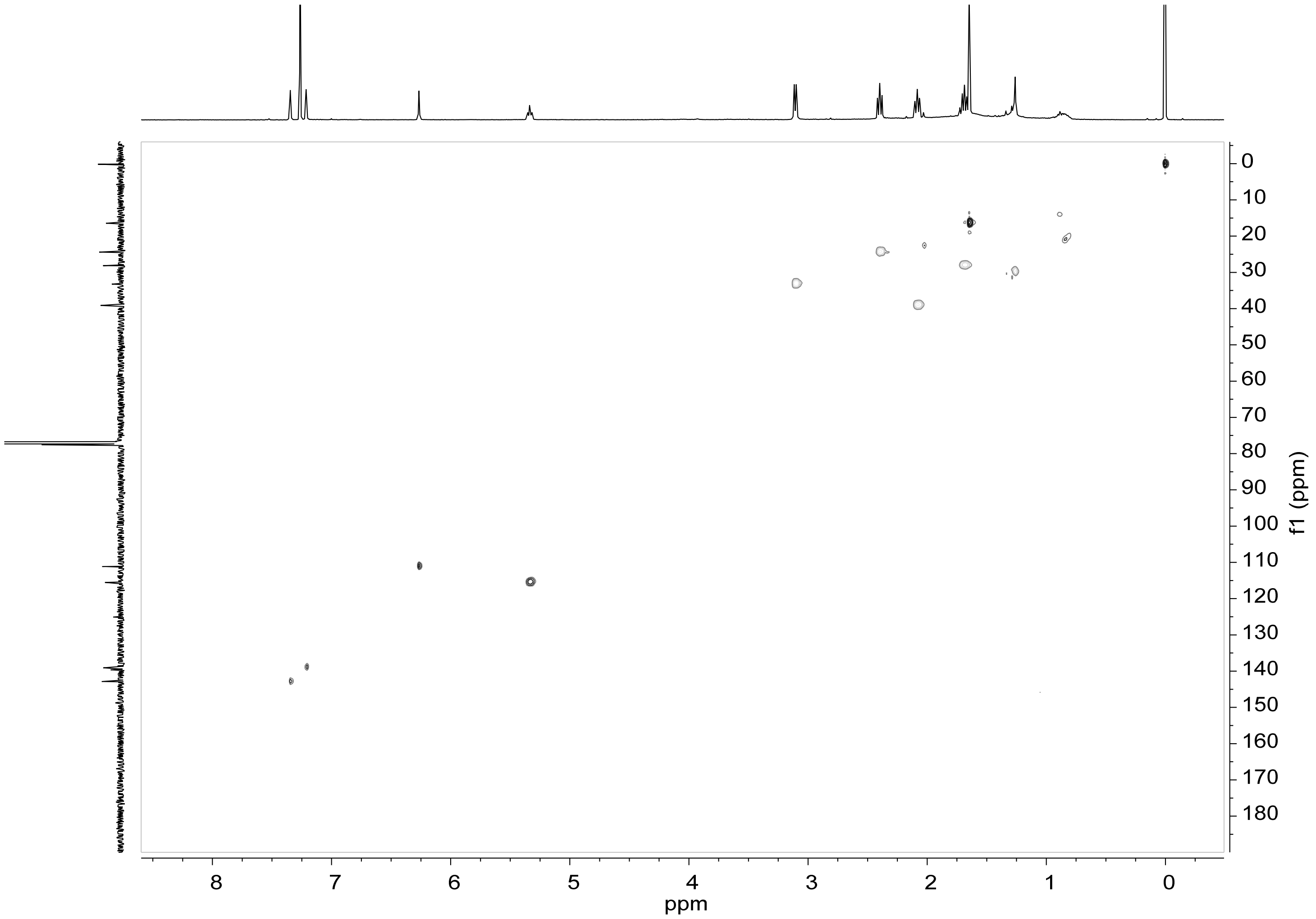

Figure S18. HSQC spectrum of **3** in $CDCl_3$.

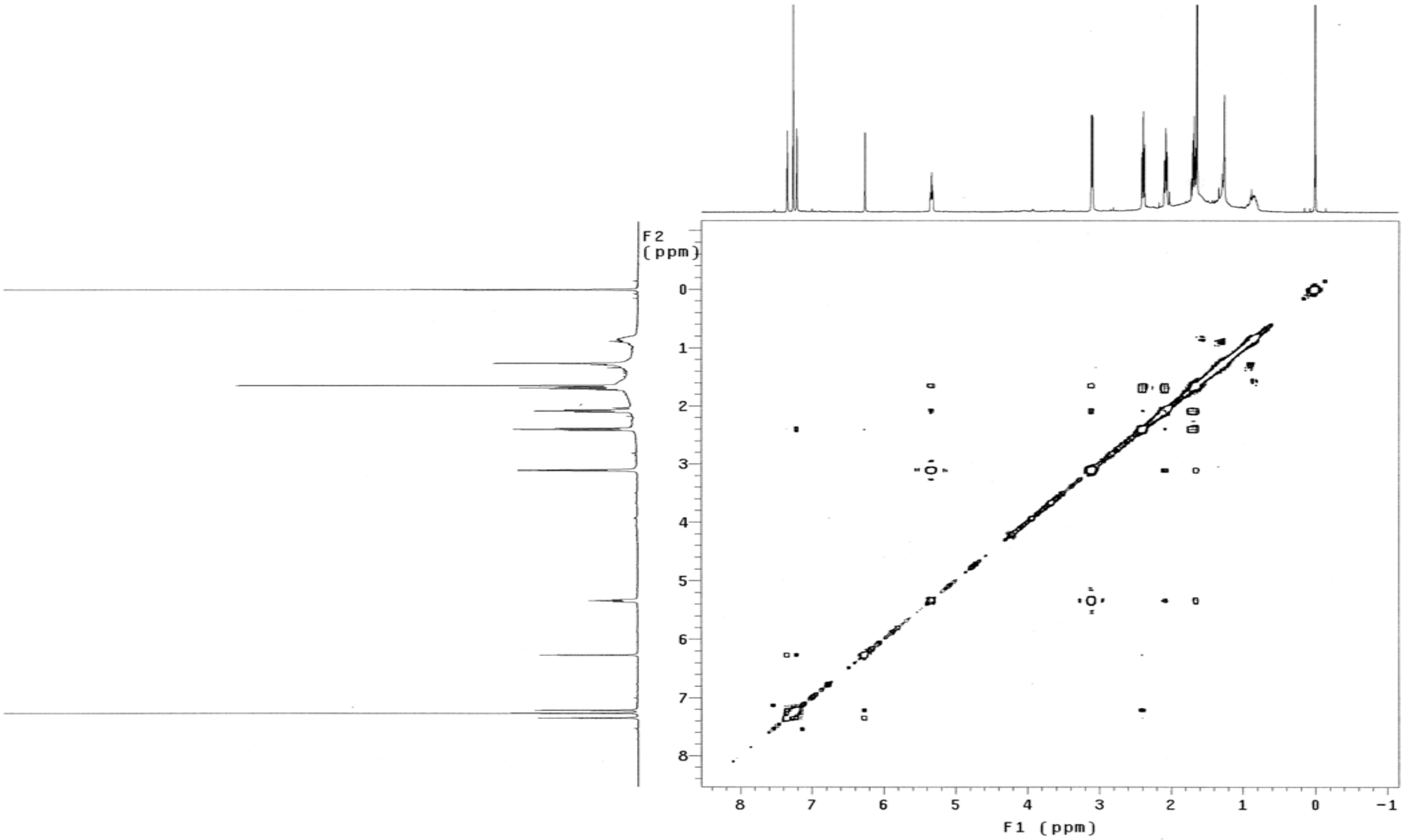

Figure S19. ^{1}H -^{1}H COSY spectrum of **3** in $CDCl_3$.

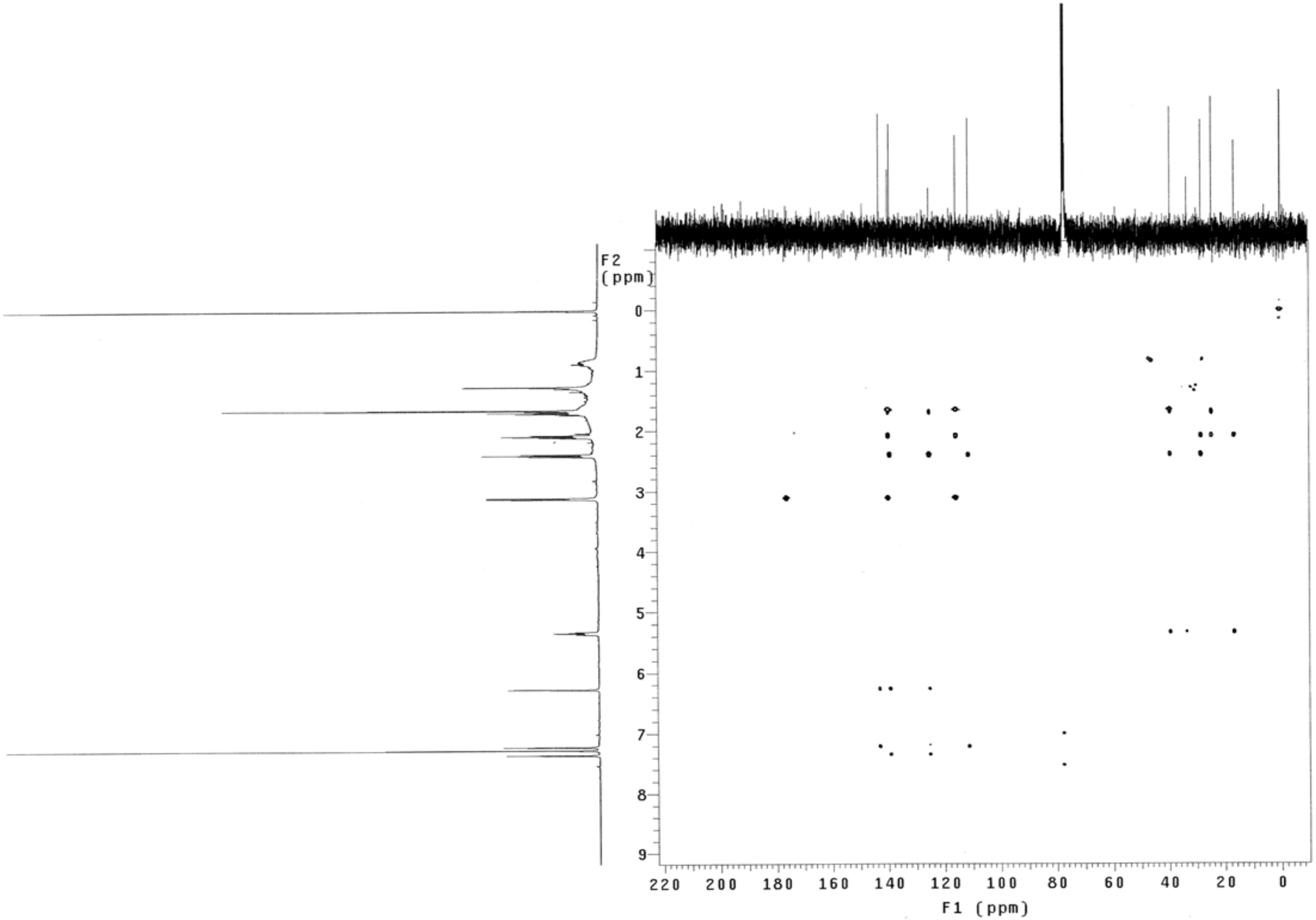

Figure S20. HMBC spectrum of **3** in $CDCl_3$.

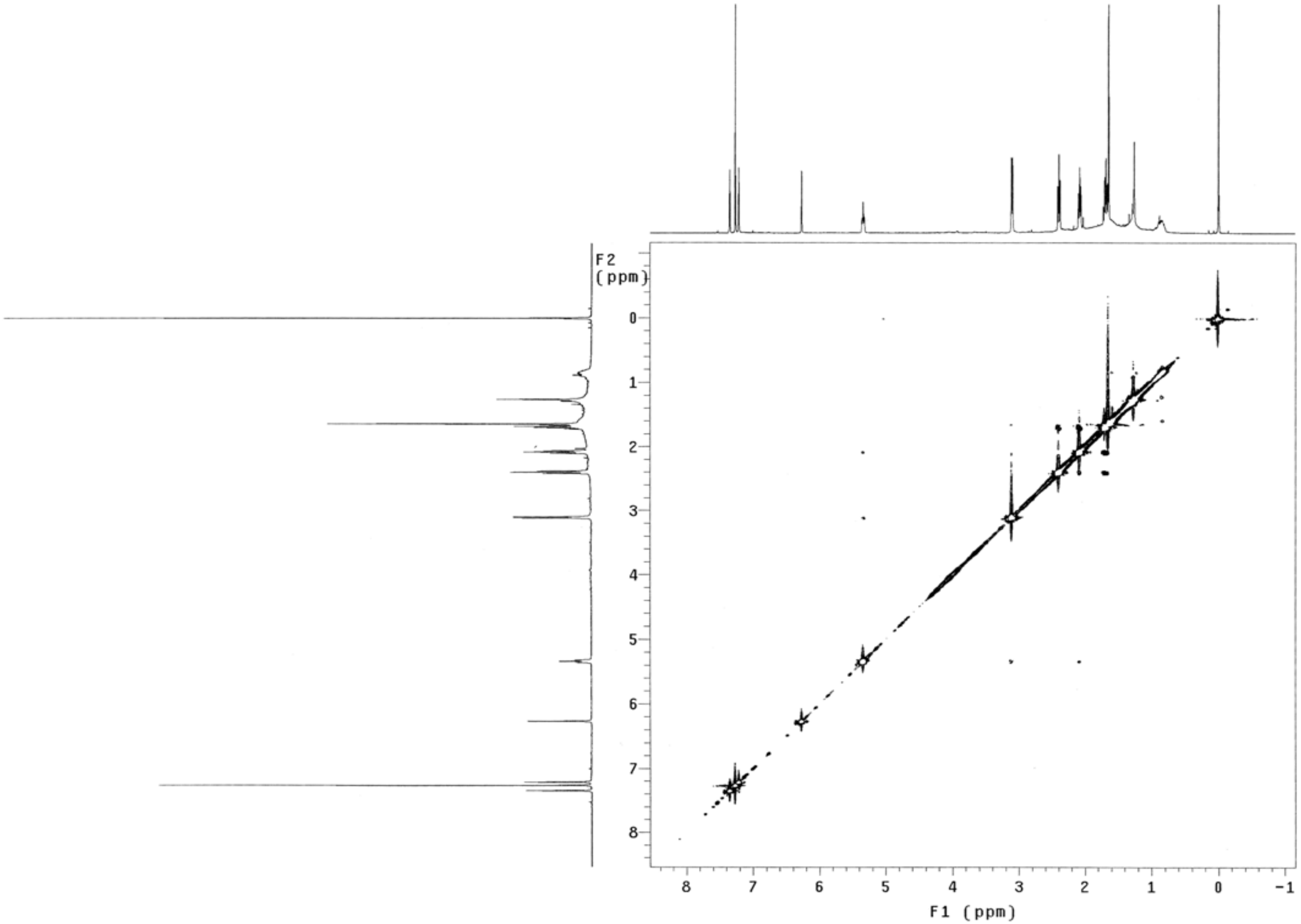

Figure S21. NOESY spectrum of **3** in $CDCl_3$.

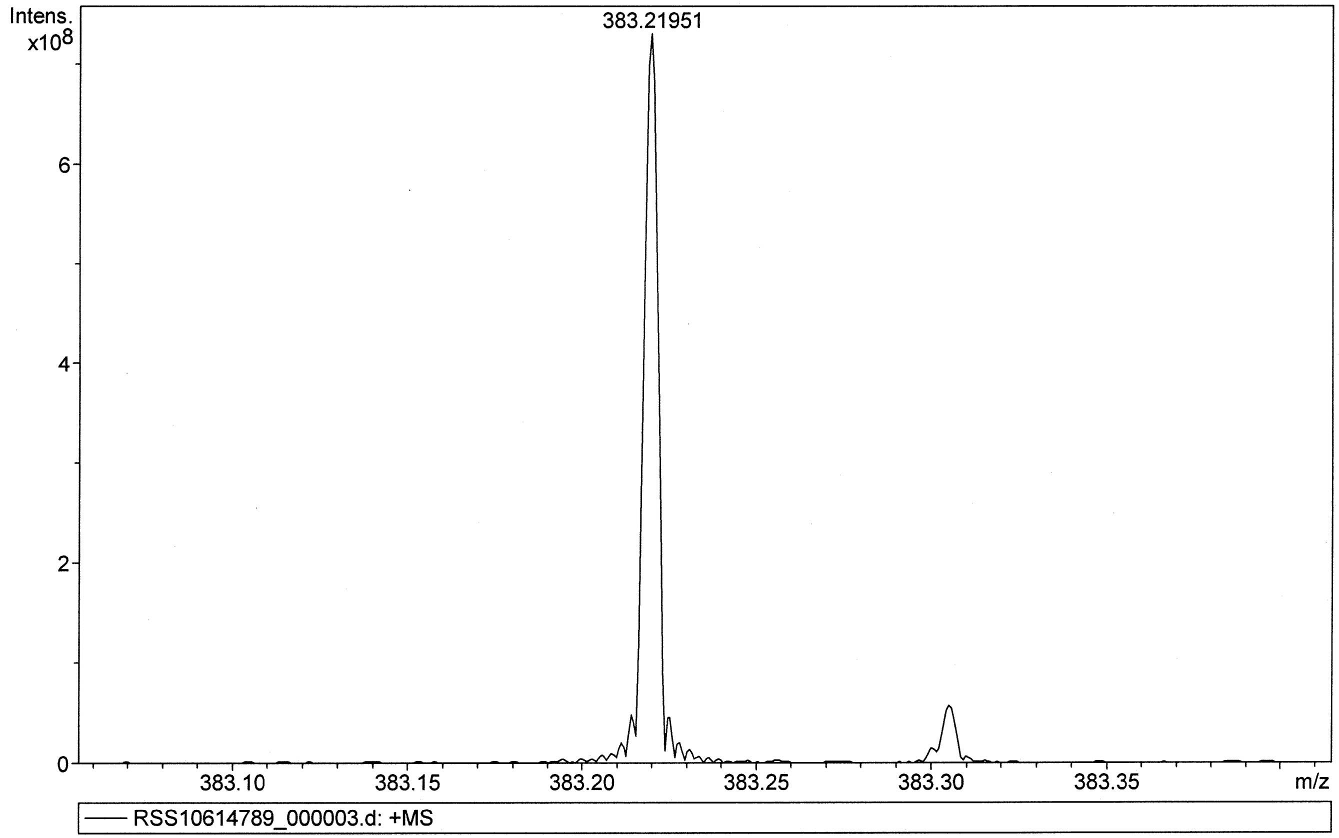

Meas. m/z	#	Formula	Score	m/z	err [mDa]	err [ppm]	mSigma	rdb	e⁻ Conf	N-Rule
383.21951	1	C 22 H 32 Na O 4	100.00	383.21928	-0.23	-0.61	8.9	6.5	even	ok

Figure S22. HRESIMS spectrum of compound **4**.

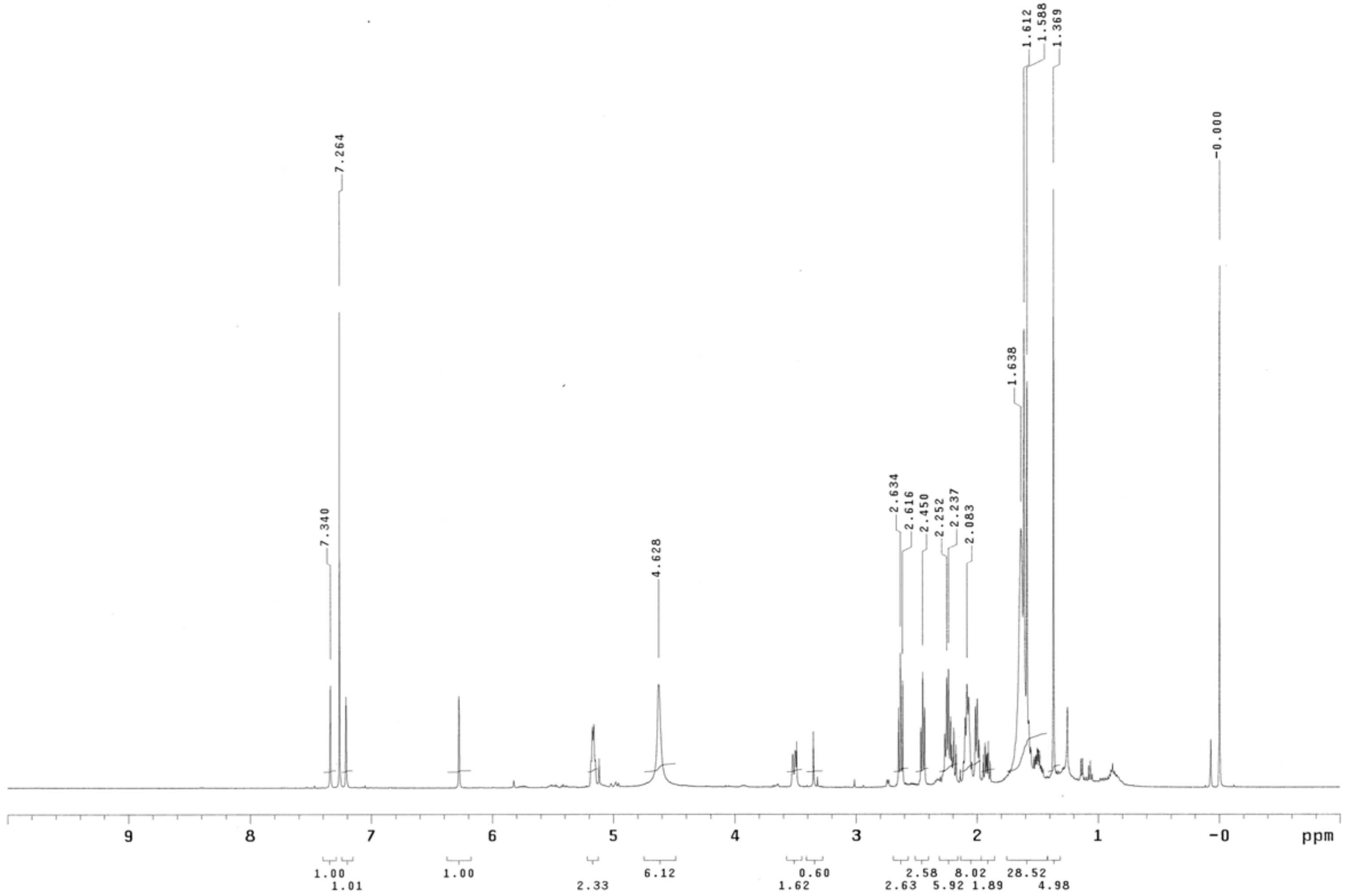

Figure S23. ^{1}H NMR spectrum (500 MHz) of compound **4** in $CDCl_3$.

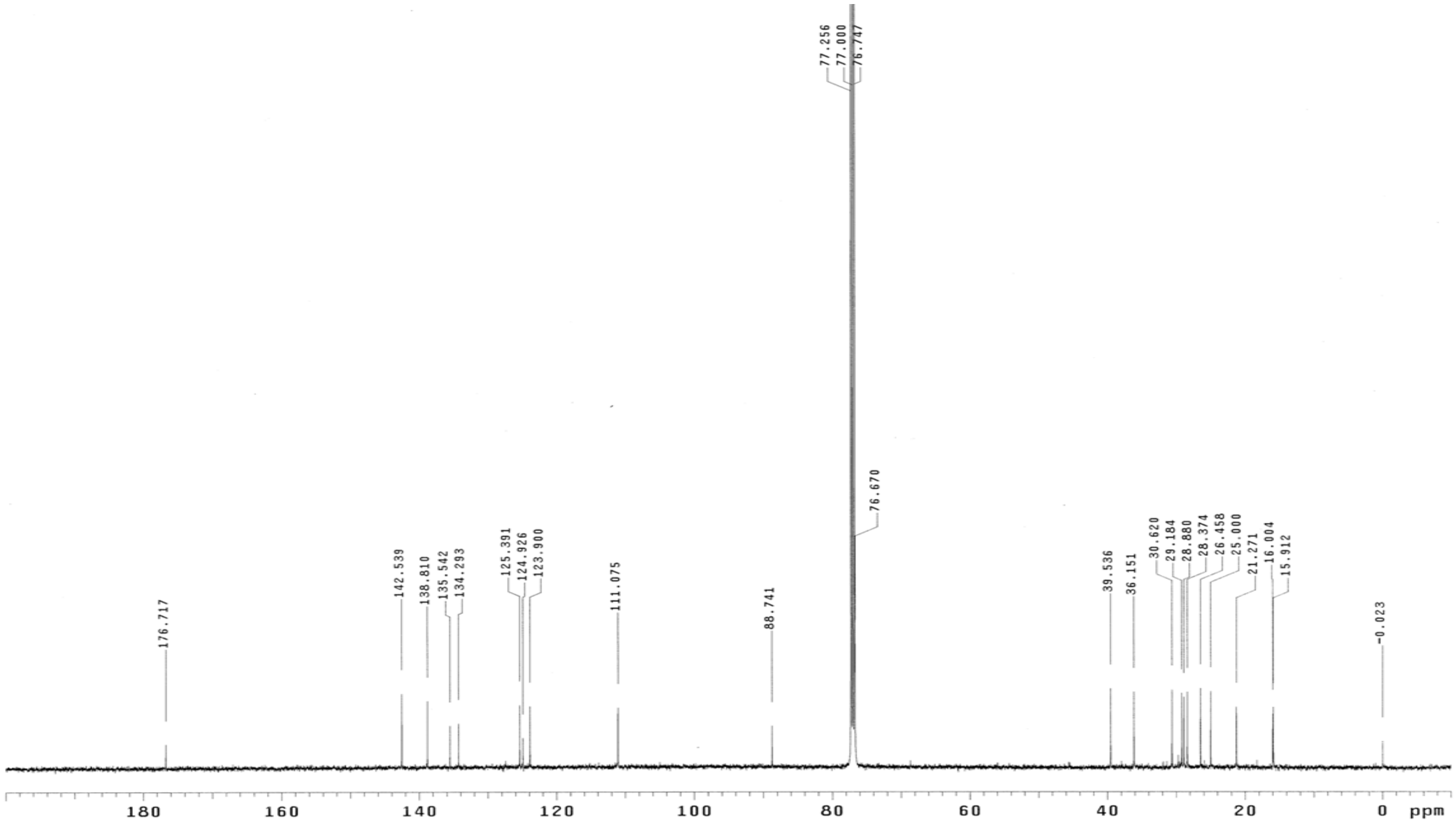

Figure S24. ^{13}C NMR spectrum (125 MHz) of compound **4** in $CDCl_3$.

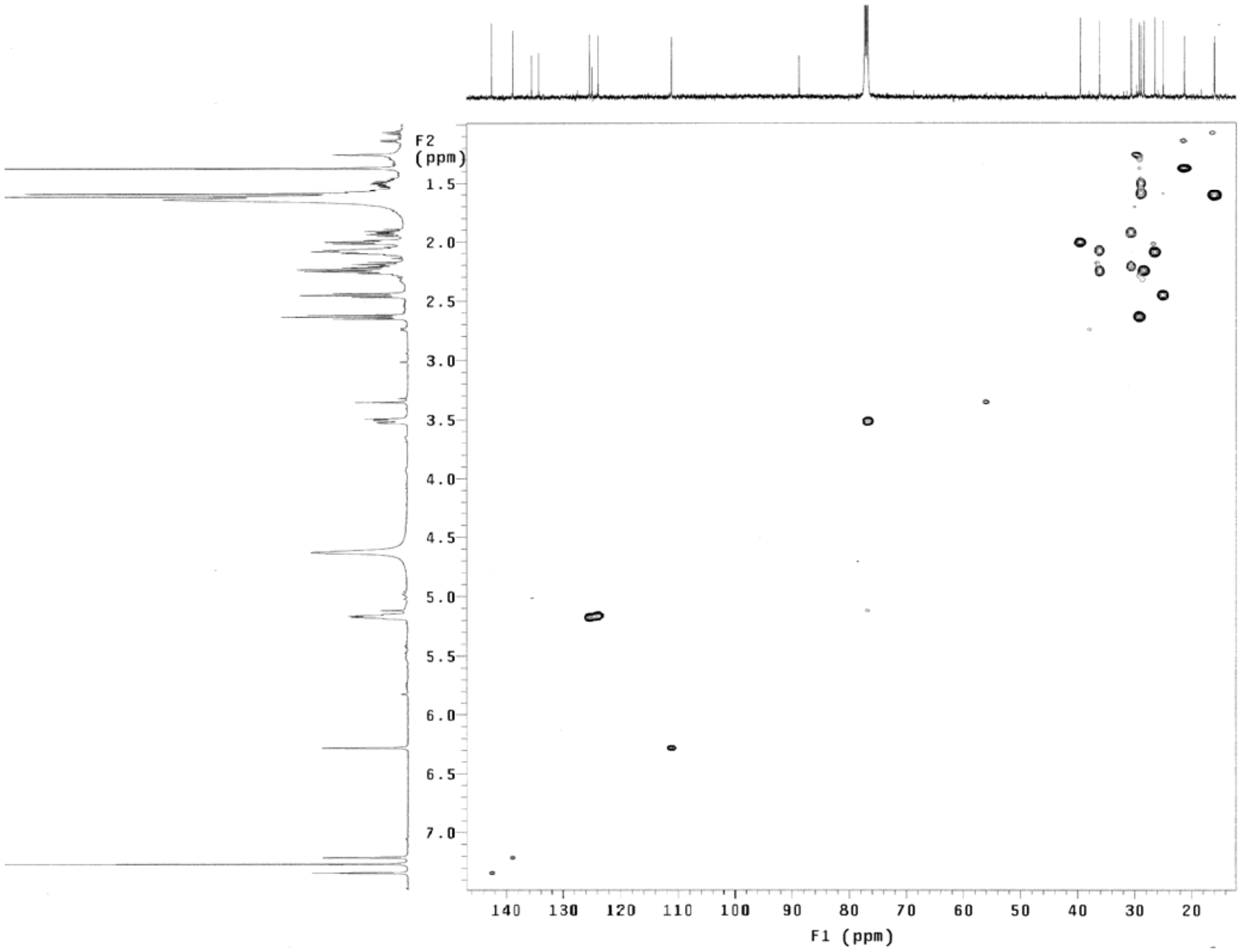

Figure S25. HSQC spectrum of **4** in $CDCl_3$.

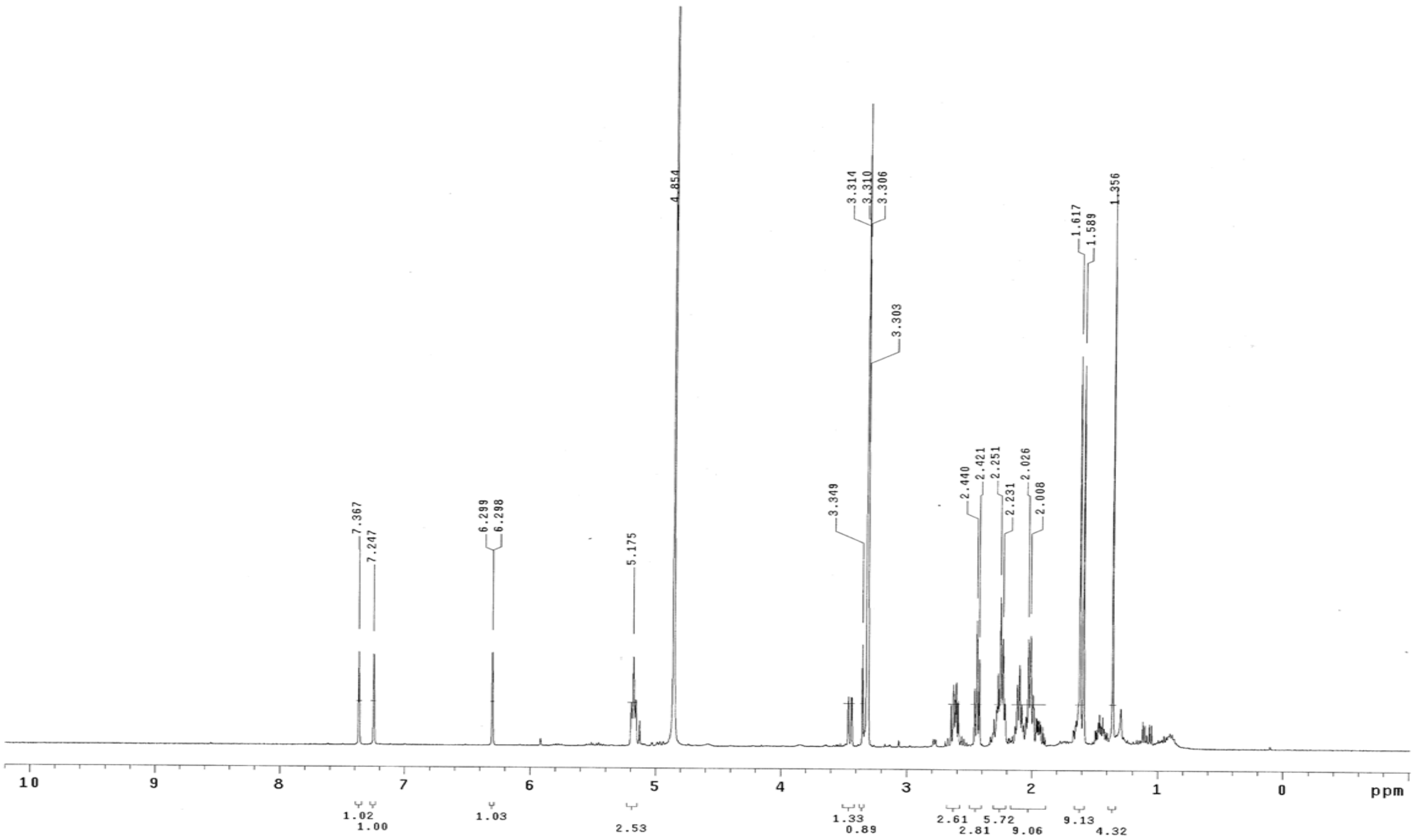

Figure S26. ^{1}H NMR spectrum (400 MHz) of compound **4** in CD_3OD.

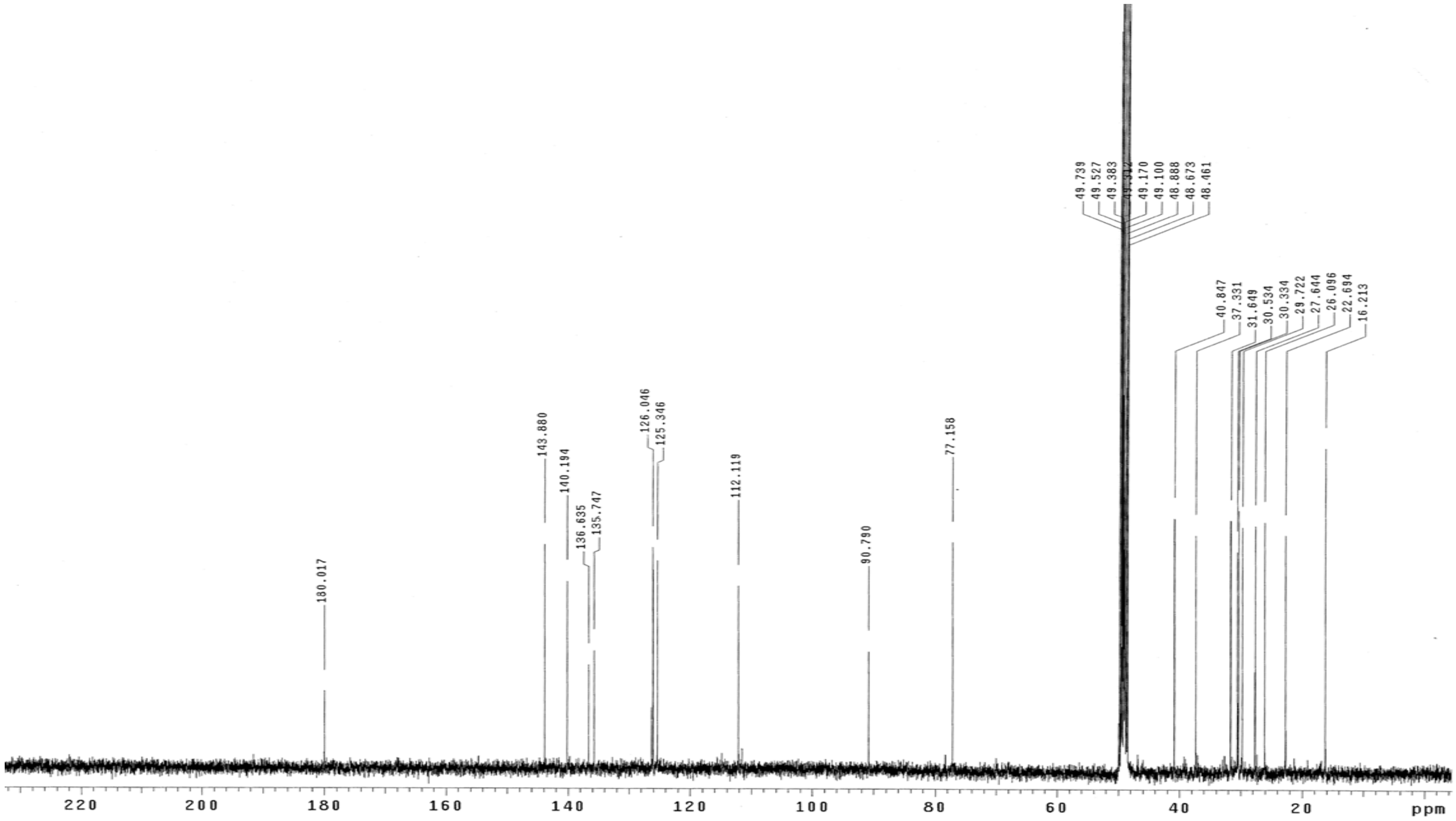

Figure S27. ^{13}C NMR spectrum (100 MHz) of compound **4** in CD_3OD.

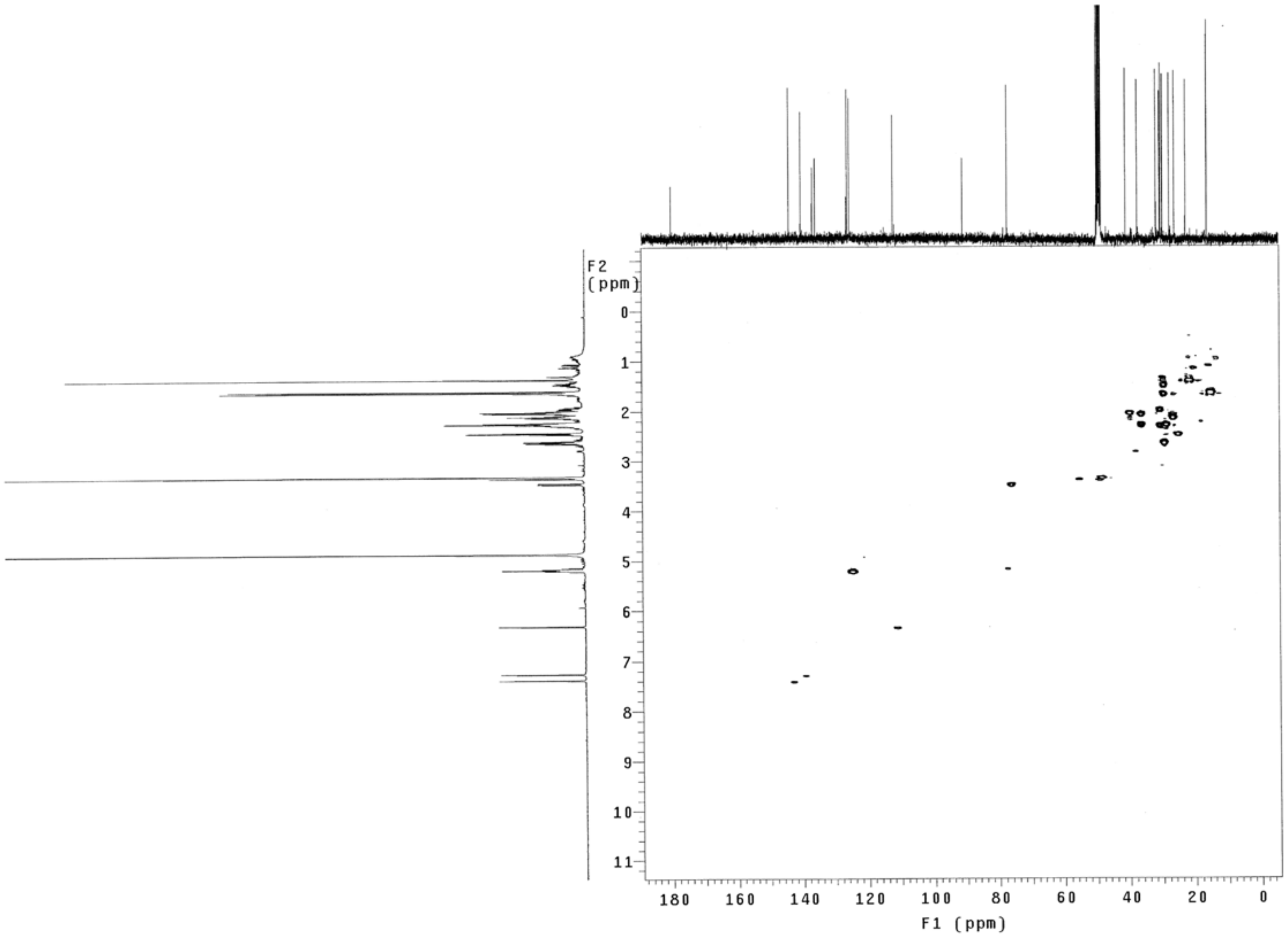

Figure S28. HSQC spectrum of **4** in CD_3OD.

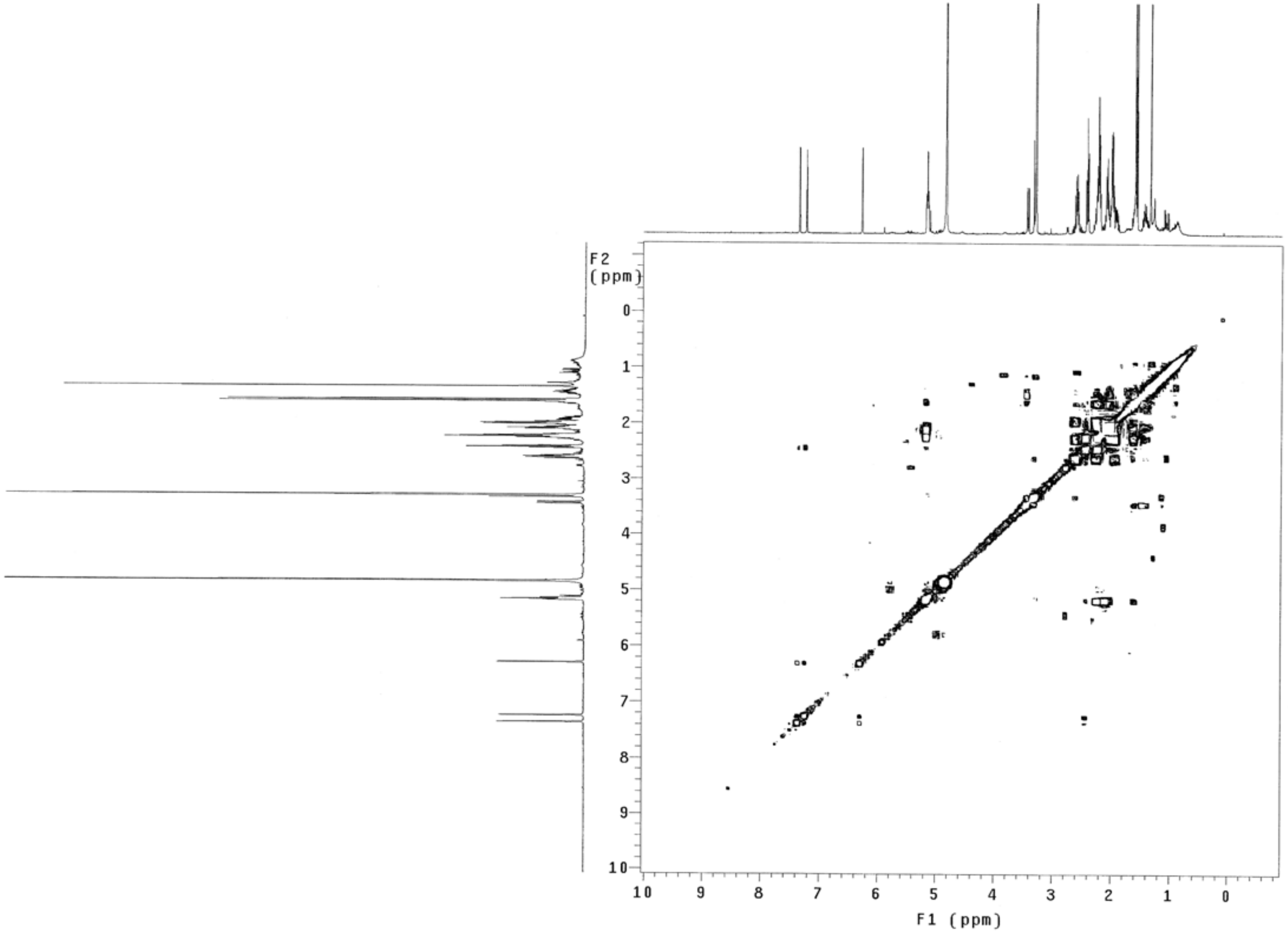

Figure S29. ^{1}H -^{1}H COSY spectrum of **4** in CD_3OD.

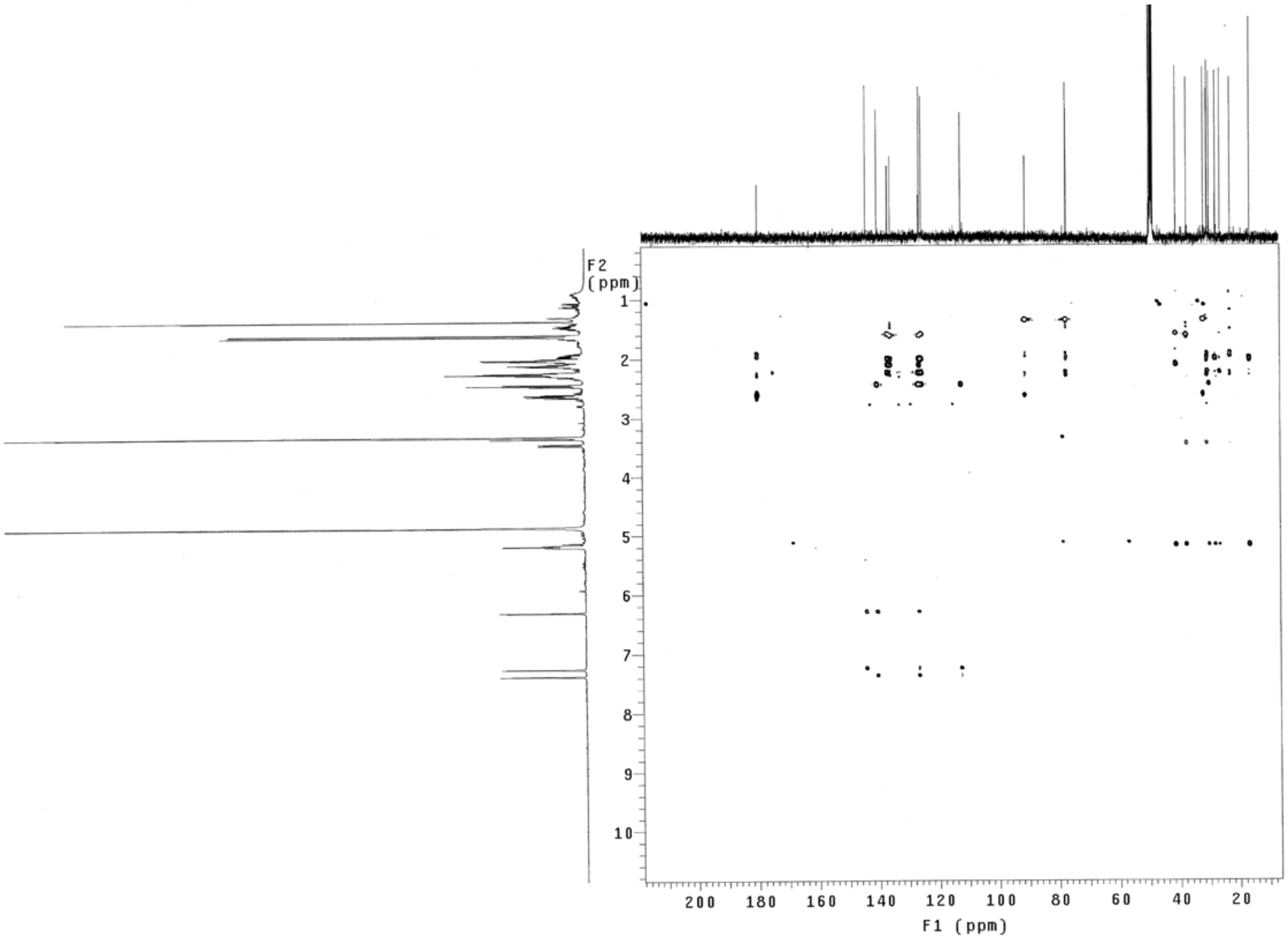

Figure S30. HMBC spectrum of **4** in in CD_3OD.

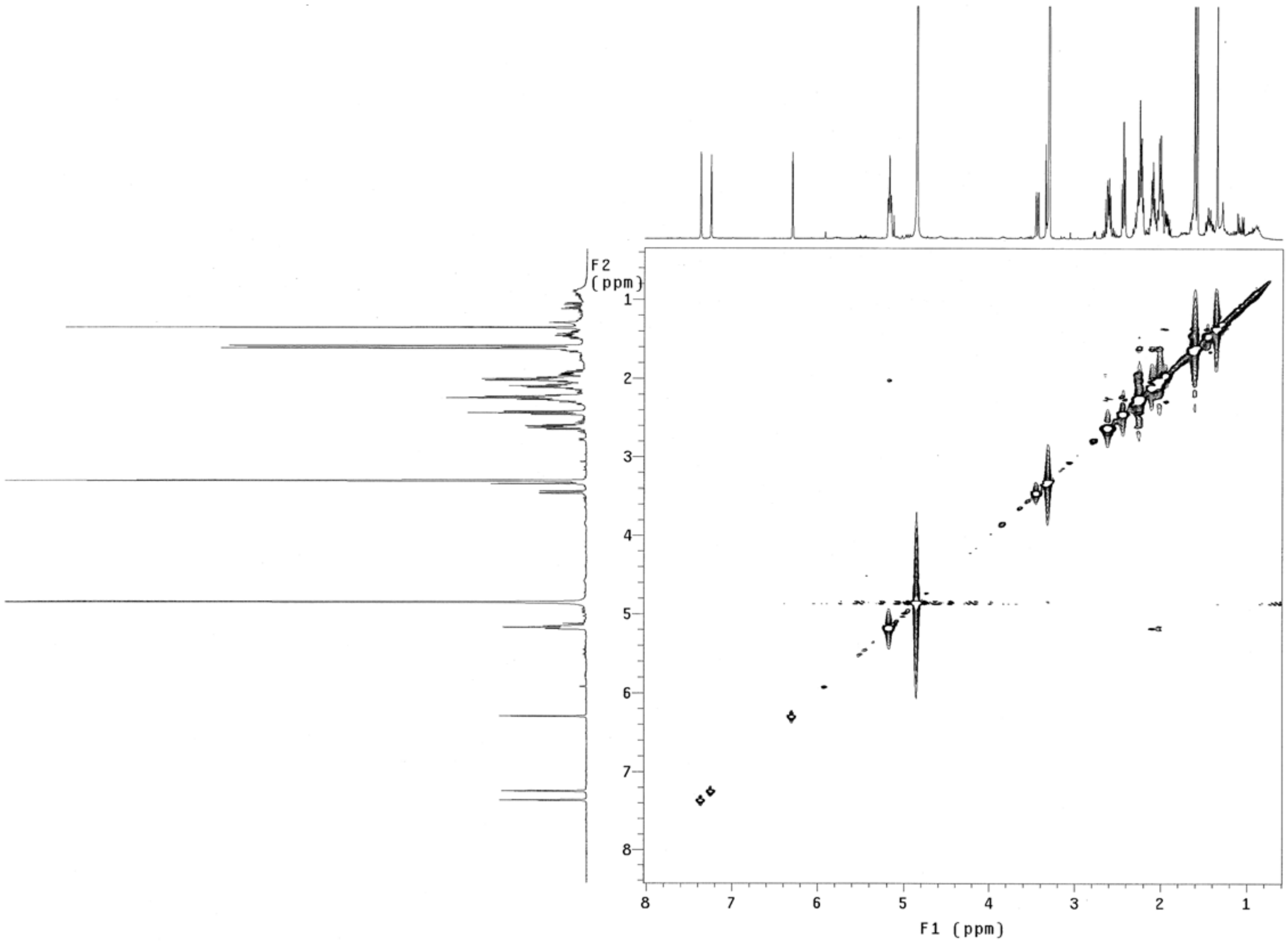

Figure S31. NOESY spectrum of **4** in in CD_3OD.

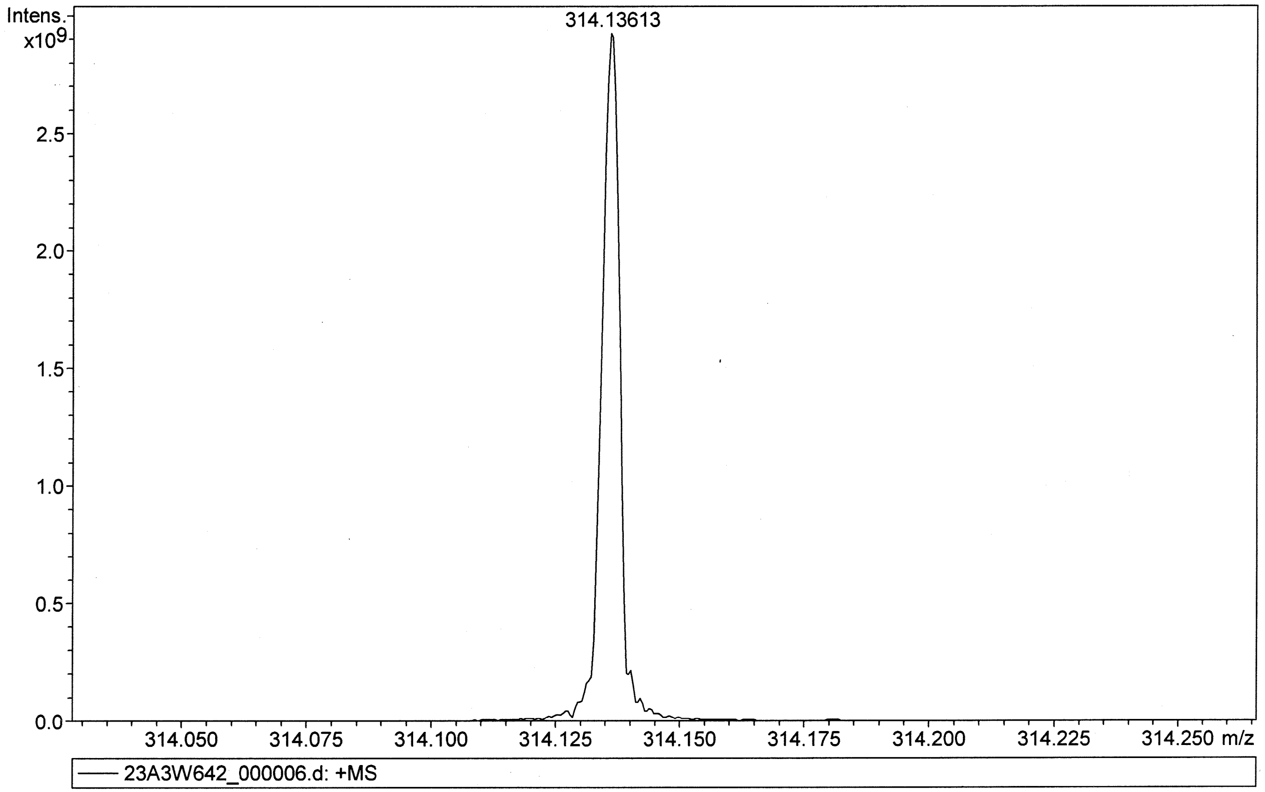

Meas. m/z	#	Formula	Score	m/z	err [mDa]	err [ppm]	mSigma	rdb	e⁻ Conf	N-Rule
314.13613	1	C 16 H 21 N Na O 4	100.00	314.13628	0.15	0.46	13.0	6.5	even	ok

Figure S32. HRESIMS spectrum of **5**.

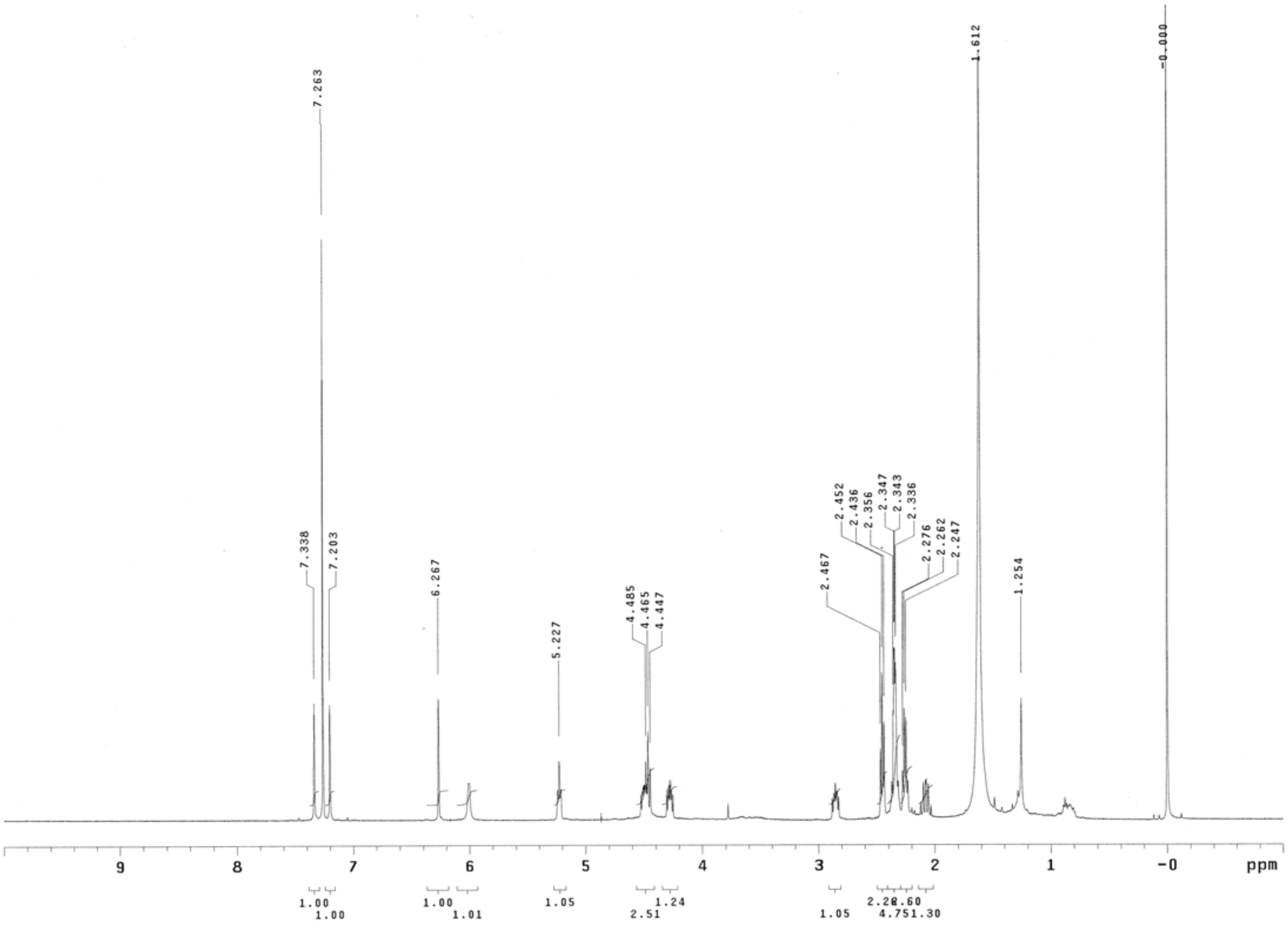

Figure S33. ¹H NMR spectrum of **5** in $CDCl_3$ at 500 MHz.

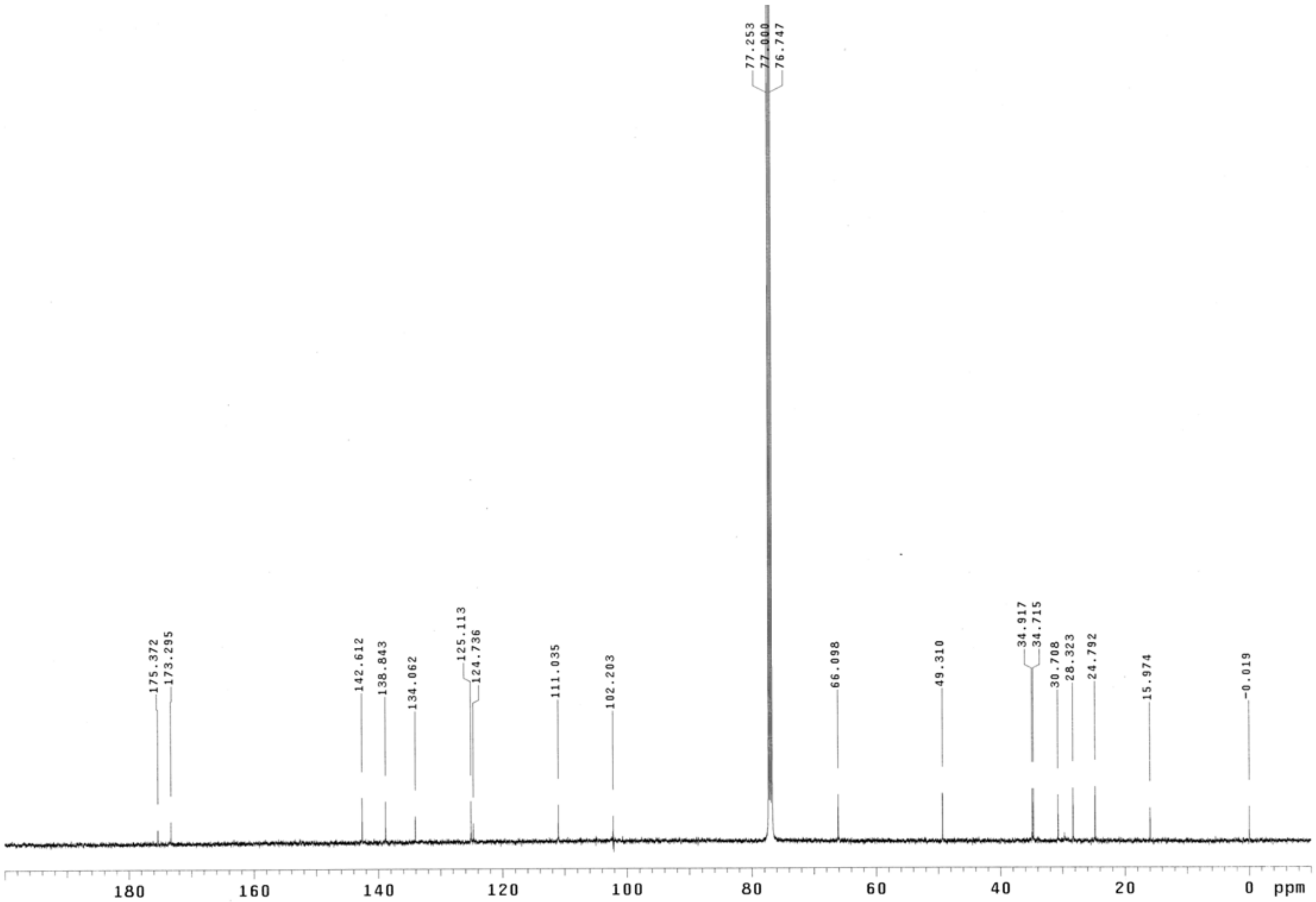

Figure S34. ^{13}C NMR spectrum of **5** in $CDCl_3$ at 125 MHz.

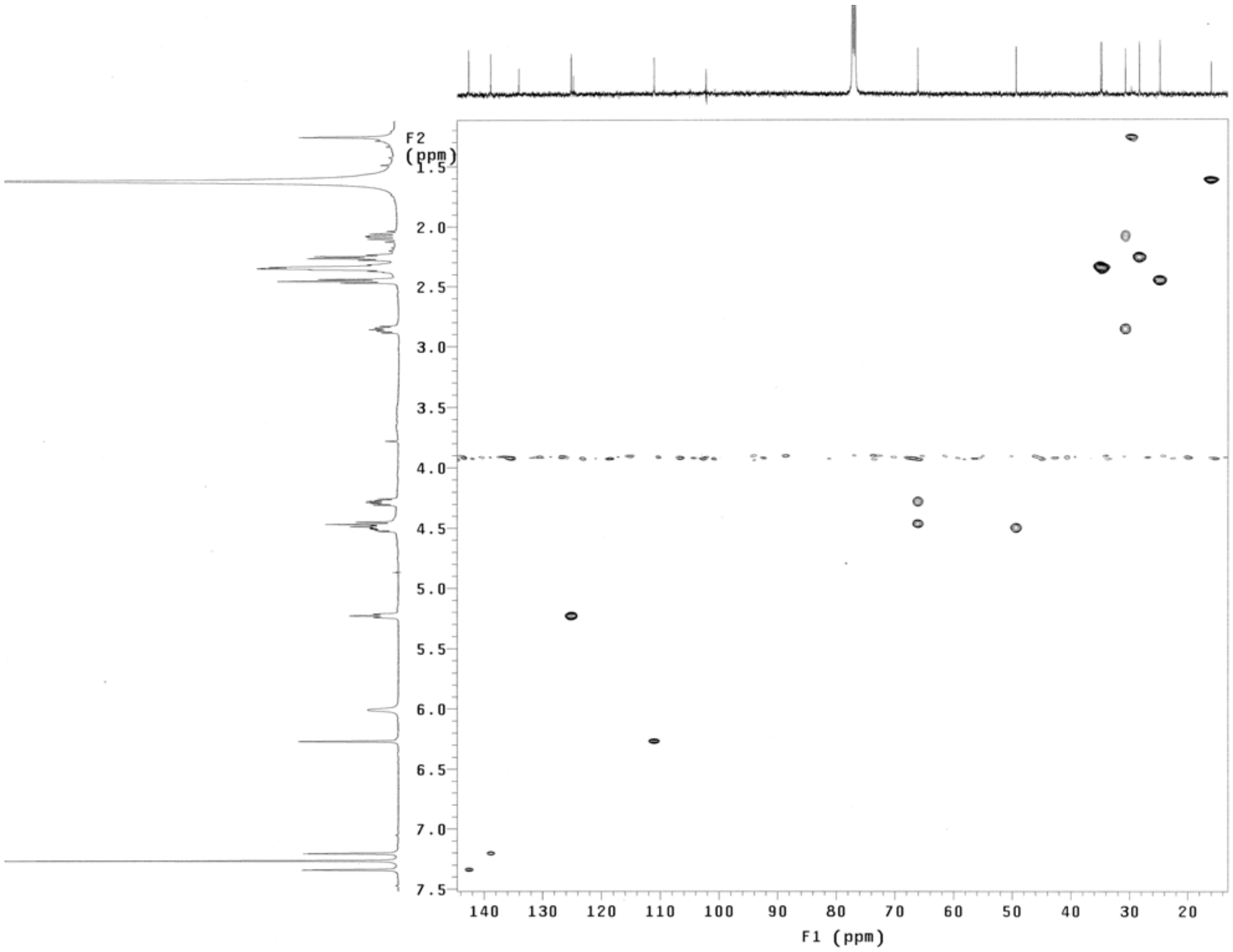

Figure S35. HSQC spectrum of **5** in $CDCl_3$.
